# Microfluidic Device for On-Chip Immunophenotyping and Cytogenetic Analysis of Rare Biological Cells

**DOI:** 10.3390/cells9020519

**Published:** 2020-02-24

**Authors:** Kumuditha M. Weerakoon-Ratnayake, Swarnagowri Vaidyanathan, Nicholas Larkey, Kavya Dathathreya, Mengjia Hu, Jilsha Jose, Shalee Mog, Keith August, Andrew K. Godwin, Mateusz L. Hupert, Malgorzata A. Witek, Steven A. Soper

**Affiliations:** 1 Department of Chemistry, The University of Kansas, Lawrence, KS 66047, USA; kratnayake@ku.edu (K.M.W.-R.); kavya87.atreya@gmail.com (K.D.); shaleemog@ku.edu (S.M.); 2 Center of BioModular Multiscale Systems for Precision Medicine, Lawrence, KS 66045, USA; swarna_vaidy@ku.edu (S.V.); larkeyn@ku.edu (N.L.); m367h922@kumc.edu (M.H.); jiljose97@gmail.com (J.J.); 3 Bioengineering, The University of Kansas, Lawrence, KS 66045, USA; 4 Department of Pathology & Laboratory Medicine, University of Kansas Medical Center, Kansas City, KS 66160, USA; agodwin@kumc.edu; 5 Children’s Mercy Hospital, Kansas City, MO 64108, USA; kjaugust@cmh.edu; 6 Biofluidica Inc., BioFluidica Research Laboratory, Lawrence, KS 66047, USA; 7 Department of Mechanical Engineering, The University of Kansas, Lawrence, KS 66045, USA

**Keywords:** microfluidics, immunophenotyping, fish, liquid biopsy, circulating leukemia cells, circulating plasma cells

## Abstract

The role of circulating plasma cells (CPCs) and circulating leukemic cells (CLCs) as biomarkers for several blood cancers, such as multiple myeloma and leukemia, respectively, have recently been reported. These markers can be attractive due to the minimally invasive nature of their acquisition through a blood draw (i.e., liquid biopsy), negating the need for painful bone marrow biopsies. CPCs or CLCs can be used for cellular/molecular analyses as well, such as immunophenotyping or fluorescence in situ hybridization (FISH). FISH, which is typically carried out on slides involving complex workflows, becomes problematic when operating on CLCs or CPCs due to their relatively modest numbers. Here, we present a microfluidic device for characterizing CPCs and CLCs using immunofluorescence or FISH that have been enriched from peripheral blood using a different microfluidic device. The microfluidic possessed an array of cross-channels (2–4 µm in depth and width) that interconnected a series of input and output fluidic channels. Placing a cover plate over the device formed microtraps, the size of which was defined by the width and depth of the cross-channels. This microfluidic chip allowed for automation of immunofluorescence and FISH, requiring the use of small volumes of reagents, such as antibodies and probes, as compared to slide-based immunophenotyping and FISH. In addition, the device could secure FISH results in <4 h compared to 2–3 days for conventional FISH.

## 1. Introduction

Molecular diagnostics are growing immensely due in part to the Precision Medicine Initiative (www.whitehouse.gov/precision-medicine), which seeks to match appropriate therapies to the molecular characteristics of a patient’s disease. Unfortunately, the majority of molecular diagnostic tests are expensive, involve slow turnaround times from centralized laboratories, and require highly specialized equipment with seasoned technicians to carry out the assay. In addition, acquisition of the molecular biomarkers requires a solid tissue or bone marrow biopsy, which can be an invasive procedure, especially for anatomically inaccessible organs. For example, bone marrow biopsies are typically required to monitor leukemia or multiple myeloma status, which not only complicates sample acquisition but limits the frequency of testing. 

Liquid biopsies are generating a significant amount of interest in the medical community owing to the minimally invasive nature of acquiring biomarkers and the fact that they can enable precision decisions on managing a variety of diseases [1,2]. Liquid biopsy markers include but are not limited to circulating tumor cells (CTCs), cell-free DNA (cfDNA), and extracellular vesicles (EVs). As an example of the utility of liquid biopsy analysis for some blood cancers, we have shown that circulating plasma cells (CPCs) can be used to stage patients diagnosed with multiple myeloma [3]. Circulating leukemia cells (CLCs) can be used to determine relapse from minimum residual disease (MRD) in patients with acute myeloid leukemia (AML) or acute lymphoblastic leukemia (ALL) [4], all of which typically require a highly painful and invasive bone marrow biopsy. The challenge with using CLCs or CPCs is that their abundance in blood is lower than what is found in the bone marrow, requiring highly sensitive assays to analyze their molecular content following enrichment to remove interfering white and red blood cells. 

A cytogenetic method called fluorescence in-situ hybridization (FISH), which was discovered in the early 1980s [5], can be used to detect chromosomal modifications [6,7,8]. FISH identifies abnormalities in chromosomes using fluorescent DNA probes that hybridize to a specific gene region. When properly hybridized to its complementary sequence, FISH allows for the visualization of chromosomal aberrations, such as deletions, fusions, balanced translocations, etc. [9]. For example, cytogenetic abnormalities are found in most cases of multiple myeloma, in which IGH translocations initiate events associated with tumorigenesis and disease progression [10]. The progression of multiple myeloma was discovered in clinical studies where investigators found frequent chromosomal aberrations, such as 13q14 deletions (del13q14), 1q21 gains (amp1q21), and monosomy 13 and 17p13 deletions (del17p13) [10,11]. B-type acute lymphoblastic leukemia (B-ALL), which is a common childhood malignancy, is prognosed by BCR/ABL [t(9;22)], MLL [t(4;1)], and TEL/AML1 [t12;21] [12] aberrations. 

While FISH assays are widely used in clinical settings, the workflow requires labor-intensive and time-consuming protocols. Conventional slide-based methods for FISH utilize workflows that necessitate the need for highly trained professionals and relatively high volumes of costly FISH probes; the full assay may require 2-3 days of processing. Therefore, it is critical to develop alternative methods and platforms to undertake FISH that address the aforementioned limitations [9,13]. 

Microfluidic FISH assays can address many of the limitations associated with slide-based FISH, such as providing process automation and reducing reagent requirements and processing time [9]. Even though there are microfluidic assays that have been developed over the past years for genetic techniques, such as PCR and DNA microarrays, less effort has been devoted toward realizing the implementation of FISH assays using microfluidic devices [14,15,16]. A summary of microfluidic devices for performing FISH are summarized in Table S1 along with their operational characteristics. 

The first microfluidic for FISH was developed by Sieben and coworkers in 2007 [17]. The study demonstrated the ability to detect a FISH signal with 10-fold higher throughput and 1/10th reagent consumption compared to slide-based FISH. Modification of the microfluidic substrate to achieve cell adherence is a common protocol to allow for processing the cells and imaging. Therefore, in this report, TiO_2_-modified glass slides were used for cell adherence to identify FISH signals, with the PDMS fluidic network used to introduce FISH reagents to the target cells. The PDMS was removed from the TiO_2_-modified glass slide, and imaging was undertaken using a 100× objective [18]. Liu et al. [19] performed FISH on centromere-sized cell arrays modified with 3-aminopropyltriethoxysilane (APTES) or polyethylene glycol (PEG)-coated glass slides with overnight hybridization. Wang et al. [20] introduced an APTES-coated glass slide with a PDMS microfluidic for stretching chromosomal DNA from a single cell to perform FISH. In a recent study, a microfluidic consisting of a Pyrex-Si stack was generated and used for FISH to provide breast cancer prognosis [21,22,23,24]. This report reduced FISH reagent consumption by 70% and hybridization time to 2 h.

Mayer et al. [24] used a microfluidic device to investigate HER2 amplification and immunohistochemistry of breast cancer patients. In a further extension of this study, the authors reported short incubation microfluidic-assisted FISH [23]. The researchers were able to reduce FISH hybridization time to 15 min for cell lines and 35 min for human tissue slides. Microfluidic channels etched into a glass slide, called FISHing lines, allowed the processing of 10 samples on a glass slide with 0.2 µL of FISH probe. MRD screening using the BCR/ABL fusion gene for chronic myeloid leukemia was performed by Mughal et al. [25]. 

Researchers have also reported using plastics to make the microfluidic for FISH to reduce the fabrication cost. Kwasny et al. [26] described the use of cyclic olefin copolymer (COC) devices sealed with glass or COC cover plates that were surface modified to allow for stretching chromosomes. Perez-Torella and coworkers reported a COC chamber, which was capable of delivering FISH reagents to cells [27]. The chamber was modified with 2-hydroxyethyl cellulose (HEC) to allow for cell adherence to the chamber walls. Micro-FISH devices fabricated with CO_2_ laser ablation in a plastic were reported, which resulted in a 20-fold reduction in the sample volume [28]. 

Our group has developed a highly sensitive microfluidic for rare cell isolation. The isolated cells were immunostained directly within the selection chip, released into a 96-well plate, and visualized/enumerated using fluorescence microscopy. In addition, following cell selection and release from the isolation chip, the cells could be subjected to FISH using conventional slide-based approaches [3,4]. Unfortunately, the workflow required extensive manual handling of cells. 

To address the workflow challenge, we developed a microfluidic device named “microtrap” for both immunophenotyping and FISH analysis of biological cells enriched from clinical samples, which we report herein. Cells isolated on the isolation chip could be transferred to the microtrap device for immunophenotyping and/or FISH in an automated fashion with reduced processing time. 

The microtrap device consisted of an array of 80,000 containment pores generated by cross-channels (2–4 µm in width and depth) connecting a network of interleaving fluidic channels. Unique to this device is that it was fabricated with containment pores or microtraps that arrayed single cells in a 2-D format and within a common imaging plane. The device does not rely on modification of the surface to retain cells; rather, it physically entraps the cells. Additionally, imaging of the cells in the microtrap device on different z-planes demonstrated better spatial resolution of the hybridization probes to designate the detection of a genetic abnormality. This feature cannot be achieved in Flow-FISH analysis [29,30]. The microtrap device provided reduced processing time (18 → 3 h) and lower amounts of FISH probes compared to slide-based processing. The microtrap device integration to a cell selection microfluidic allowed for automated cell processing as well as minimized the amount of operator handling of the enriched cells, simplifying the workflow and reducing cell loss. 

We demonstrate the utility of the device for the processing of non-adherent cells, such as CPCs and CLCs, with the device being able to perform immunophenotyping and FISH from liquid biopsy markers. In addition, we demonstrate that we can affinity-enrich B-ALL CLCs from a pediatric patient blood sample using a cell enrichment microfluidic decorated with anti-CD19 antibodies and perform FISH and immunophenotyping on the enriched cells using the device reported herein. 

## 2. Materials and Methods

### 2.1. Design and Fabrication of the Microtrap Device

**Figure 1:**
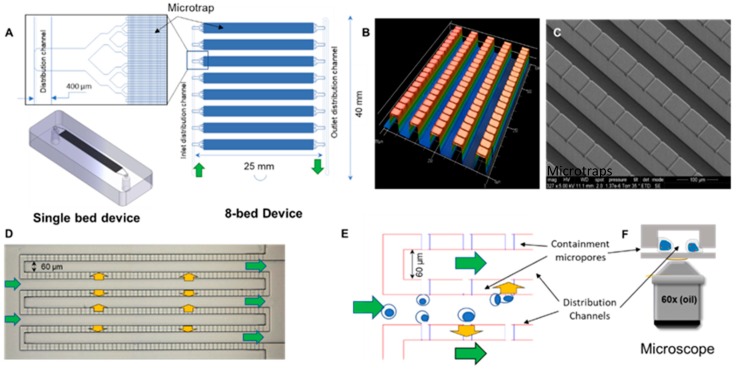
Microfluidic device for performing automated immunophenotyping and FISH. (**A**) Design of the microfluidic network composed of a single bed with 7200 microtraps and the 8-bed device containing 10,000 microtraps in each bed for a total of 80,000 traps per device. Microtrap size: 4 × 2 × 50 µm (w × d × l). (**B**) Profilometer scan of the microtrap chip replicated in PDMS from a 3-level SU-8 relief and a Si master showing microchannel depth varying between input/output distribution channels, interleaving channels, and cross-channels. (**C**) Cross-channels and the deeper interleaving channels are shown in the SEM image. (**D**) Optical microscope image of a lithographically patterned 2-level SU-8 relief for preparing a single bed microtrap device. The arrows show the fluid path. (**E**) Schematic showing operation of the microtrap chip. Cells in solution (green arrows) are contained at the entrances of the microtraps, letting the fluid pass (yellow arrows) into the outlet channels of the interleaving network. (**F**) Schematic showing the 3-dimensionality of cells captured in the microtrap chip and imaging using a high magnification (60× or 100×) objective through a thin cover plate.

The design of the microtrap device is shown in Figure 1. There are two basic renditions, with each differing in terms of the number of microtraps the device possessed. Design 1—single bed with 7200 microtraps; and Design 2—8-bed device with 80,000 microtraps. The microtrap size could vary depending on the width and depth of the cross-channels and was as small as 4 µm (width) × 2 µm (depth) to accommodate the containment of smaller cells, such as CPCs and CLCs (6–16 µm in diameter), compared to the larger-sized CTCs [31]. The 8-bed device (shown in Figure 1A,B) consisted of a significantly increased number of microtraps as compared to our previous design due to the high numbers of cells that are enriched for leukemia and multiple myeloma diseases compared to CTCs’ affinity enriched from epithelial cancers [3,4,31].

The lithography and fabrication steps of both devices are discussed in detail in the Supplementary Materials. The architecture of the single bed device (2-step lithography to create two levels) consisted of two independent networks of interleaving channels that were 60 × 40 µm^2^ (W × D) and interconnected using an array of cross-channels orthogonally placed in between the interleaving channels (see Figure S1). The volume of this device was ~1 µL. For the 8-bed device, 3-level lithography was used to allow generation of a deep layer for the distribution channels, interleaving channels, and cross-channels (see Figure S2). Profilometry scans of the 8-bed chip showed the 3 levels for the SU-8 relief (Figure 1B). Figure 1C shows an SEM of the lithographically patterned 2-level SU-8 relief of a single bed device, with a magnified section showing the replica PDMS device with interleaving channels (30 μm) and shallow cross-channels (2–4 μm). In the 8-bed device, the 3 rd lithography layer was used to fabricate a deeper distribution channel network to distribute fluid to all 8 beds equally [32]. The distribution channels of the 8-bed device were 400 x 150 µm (W × D). The internal volume of this device, including all fluidic channels, was ~10 µL. 

The fluidic operation of the device is shown in Figure 1D,E, where the flow from the interleaving channel input flows across (90° angle) the interconnecting cross-channels and then to the output channels of the interleaving network. The cross-channel, when a cover plate was bonded to the chip substrate, generated a microtrap structure where cells were contained but still allowed fluid to flow through it. 

The optimal device design was chosen from testing 14 different designs for which we determined the containment efficiency of CPCs and CLCs. The 14 devices with different pore widths (4, 6, and 8 μm) and different depths (2 and 4 μm) were evaluated in terms of their ability to trap CPCs and CLCs (data not shown). The schematic in Figure 1D shows how the cells were contained at the entrance of the microtraps and high magnification imaging of the trapped cell as shown in Figure 1E. 

An SU-8 relief was used to prepare PDMS trapping devices that were bonded to glass cover plates to allow for high resolution fluorescence imaging. Reservoirs were formed in the PDMS device using a sharp biopsy puncture. PDMS devices and glass cover plates (No.1 coverslips with a thickness of 0.13–0.16 mm) were cleaned with IPA, then washed with water, and air dried. We used thin glass coverslips to facilitate the use of high NA objectives to accommodate their short working distances and high magnification necessary for FISH. PDMS devices were bonded to the glass coverslips by treating the surfaces with O_2_ plasma (50 W for 1 min). After plasma treatment, slight pressure was applied starting from one edge to the other to avoid the trapping of air bubbles. Peak tubing was sealed with epoxy glue to the reservoirs and a syringe pump was used to provide continuous fluid flow through the device.

### 2.2. Sample loading 

The microtrap device was flooded with a continuous flow of 25 μL/min of PBS buffer (pH 7.4, 100 mM) followed by flowing 0.5% BSA/PBS solution through the device using a PHD2000 syringe pump (Harvard Apparatus, Holliston, MA, USA). Forty μL/min flow rates were used to remove air bubbles from the device. Once the device was fully wetted with the 0.5% BSA/PBS solution, cells were introduced into the device for either immunophenotyping or FISH at a volume flow rate of 10 μL/min. 

### 2.3. On-Chip immunostaining 

For immunostaining, live cells (RPMI-8226 or SUP-B15) were loaded as described above onto the microtrap device. After loading target cells onto the device, 2% paraformaldehyde (PFA) was injected at 10 μL/min for 2 min and allowed to incubate for 15 min. After incubation, the device was washed with PBS buffer for 5 min. Fixed cells were then treated with human Fc blocker (IgG1) for 15 min to block any Fc receptors on the cell surface followed by incubation with monoclonal antibodies for 30 min. For RPMI-8226 cells, anti-CD138 FITC (MI15, 5.0 μg/mL), anti-CD56-PE (MEM-188 clone, 20 μg/mL), and anti-CD38-APC (HIT2 clone, 2.5 μg/mL) monoclonal antibodies were used. Cells were then washed with PBS and permeabilized with 0.1% Triton X-100 for 5 min followed by counter staining with DAPI for 2 min. 

### 2.4. Sample Preparation for FISH

RPMI-8226 cells were cultured as a model CPC cell line for multiple myeloma and similarly, a SUP-B15 cell line was cultured as a model CLC for B-ALL (see Supplementary Materials for more information). Once the cells were removed from the culture media, they were washed with 1× PBS twice. Following the wash, the cells were re-suspended in 0.075 M KCl hypotonic solution plus 100 µL of Colcemid to swell the cells. Colcemid helps the chromosomes to stretch, thus enhancing the clarity and the resolution of the fluorescent probes used for FISH. Cells were fixed using Carnoy’s fixative (methanol: glacial acetic acid = 3:1 (*v*/*v*)). Carnoy’s solution is a light fixative (no crosslinking) as opposed to 2% PFA. During fixation, Carnoy’s fixative was added dropwise to the cells and mixed with gentle agitation. The solution was then centrifuged at 1200 rpm for 10 min. This step was repeated 3 times and the fixed cells were stored at −20 °C until being used for experiments.

### 2.5. On-Chip FISH

Before using fixed cell samples prepared as described above, they were mixed with fresh Carnoy’s solution, and introduced into the microtrap device at 10 μL/min. In the case of live cell samples, cells were fixed on-chip before processing for FISH. Once the live cells were injected and contained at the microtraps in the device, 0.056 M KCl hypotonic solution was injected and cells were incubated for 10 min. After KCl treatment, cells were fixed by injecting Carnoy’s fixative and incubated for 30 min, replacing the solution with fresh Carnoy’s every 10 min. Then, all of the Carnoy’s solution was removed by washing with PBS. 

Next, the chip was washed with 2X SSC for 2 min at room temperature followed by a series of ethanol washes (70%, 85%, and 100% EtOH) injected for 1–2 min each and dried with 100% EtOH for 5 min. After the EtOH wash, the chip was dried completely by heating and evaporating all of the EtOH. One μL and 10 μL of 5× diluted FISH probe mix was introduced into the single and 8-bed device, respectively, while applying light vacuum to the outlet tubing. Once the device was filled with FISH probes, the inlet and outlet were sealed with rubber cement. The chip was heated to 75 °C for 5 min and incubated in a hybridization oven (Bambino II™ Hybridization Oven) (Boekel Scientific, Feasterville, PA, USA) for 2 h at 37 °C. Afterwards, the rubber cement was removed, and the chip was kept at 72 °C (±1 °C) while washing for 2 min with 0.4X SSC (pH 7.0). The temperature of this step is critical as it will remove the remaining free probes and keep hybridized ones associated to the target DNA to improve imaging of the FISH signal. Next, the device was washed with 2 × SSC + 0.05%, and Tween-20 (pH 7.0) for 1 min at room temperature. Finally, the device was washed with EtOH for 5 min and dried completely before applying 2 μL of 4-diamidino-2-phenylindole (DAPI II) for nuclear staining. After these steps, the chip could be stored at 4 °C until imaging. 

### 2.6. Imaging On-chip and Image Processing 

All imaging was performed using a Keyence BZ-X710 microscope (Keyence Cooperation of America, Itasca, IL, USA) equipped with BZ-X filters; DAPI (Ex: 360/40 nm, Em: 460/50 nm, dichroic mirror wavelength (DMW): 400 nm), GFP (Ex: 470/40, Em: 525/50 nm, DMW: 495 nm), TRITC (Ex: 545/25 nm, Em: 605/70 nm, DMW: 565 nm), and Cy5 (Ex: 620/60 nm, Em: 700/75 nm, DMW: 660 nm). The microscope was equipped with a Nikon objectives (Nikon Instruments, Melville, NY, USA) CFI Plan Apo 4× (λ 4×, NA 0.20 WD 20.00 mm), 10× (λ 10×, NA 0.45 WD 4.00 mm), 20× (λ 20×, NA 0.75 WD 1.00 mm), 40× (λ 40×, NA 0.95 WD 0.21 mm), and 60× (Apo VC 60× NA 1.40 NA WD 0.13 mm) objectives. For immunophenotyping, exposure times of 50 ms for DAPI, 500 ms for FITC, and 1500 ms for TRITC/Cy3 and Cy5/APC were used at 10×, 20×, and 40× magnification. All images for FISH experiments were acquired in high-resolution mode. Cell nuclei were imaged with the 60× objective using DAPI (blue) filters (200 ms) and the FISH signals were acquired using FITC/GFP (green, 1500 ms) and (Cy5/APC)/Cy3 (red, 2500/3000 ms) channels. Due to the cell’s 3-D profile while they were contained at the microtrap inlets, it was necessary to do imaging across the *z*-axis. For the imaging of slides, we used Δz = 2 μm for 5 different planes while for on-chip FISH imaging, cells were imaged at 10 different planes along the *z*-axis with Δz = 1 μm. All images were processed using BZ-X Analyzer (Keyence Cooperation) and FIJI software (NIH) [33]. 

### 2.7. Patient Sample Processing for FISH

CLCs from B-ALL patients were captured using the CTC isolation device described in our earlier publications (see Supplementary Materials for more information), which was also used for acute myeloid leukemia (AML) and multiple myeloma work [3,4]. Healthy donor blood sample was obtained from the University of Kansas Medical Center (KUMC) IRB-approved Biospecimen Repository Core Facility. Blood samples from a patient diagnosed with B-ALL was collected according to an approved Children’s Mercy Hospital Institutional Review Board procedure. Written informed consent was obtained from the patient included in the study before enrollment. Peripheral blood samples (5 mL) were drawn by venipuncture into Vacuette^®^ K3EDTA (Greiner Bio-one, Monroe, NC, USA) tubes. Following affinity enrichment using the microfluidic enrichment chip, cells were released from the capture device’s surface using USER enzyme that cleaved a single-stranded bifunctional linker containing a uracil residue [34]. The released cells were collected into a microfuge tube and centrifuged to prepare for immunophenotyping or FISH. For FISH, once the cells were spun down, supernatant was removed and pre-heated (37 °C) in 0.056 M KCl and incubated for 10 min. The mixture was centrifuged again and after removing the supernatant, ice-cold Carnoy’s fixative (if fixation was done before introduction to the microtrap device) was added to the cells and centrifuged again. This step was repeated 3 more times and cells were stored in Carnoy’s fixative at 20 °C until further use. CLCs were spun down and resuspended in fresh Carnoy’s solution before use. These samples were infused into the microtrap device and processed according to the on-chip FISH procedure as described above.

## 3. Results

### 3.1. Microtrap Device for Immunophenotyping and Cytogenetic Analysis

The large number of traps in the microtrap device needed to be contained for analysis of CLCs’ and CPCs’ affinity selected from a blood sample (see Table 1). Contrary to CTCs, which use, for example, EpCAM as the enrichment antigen, the number of non-diseased cells selected by antibodies targeting leukemic associated and multiple myeloma antigens is high because non-diseased cells can also express the enrichment antigen (i.e., CD19, CD34, CD117, CD33, or CD138). Following CLC or CPC affinity selection and release from the selection chips [3], cells were trapped without surface modifications at containment pores of the microtrap device, which were arranged in a 2-D format to make it easier for imaging single cells. This simplified imaging was compared to cells stochastically arranged on glass slides or wells possessing the appropriate surface chemistry. In addition, we will show that we can interface the microtrap device for immunophenotyping and FISH of CLCs and CPCs to a device used for their enrichment from the blood of patients. 

Considering the size of the CLCs and CPCs with an average diameter of 6–16 µm, we used 4 × 2 µm^2^ microtraps to maximize the containment efficiency of CLCs and CPCs. Table 1 shows the number of CLCs for both AML and B-ALL as well as CPCs enriched using the appropriate marker(s). The numbers of CTCs enriched from different epithelial cancers (i.e., pancreatic, breast, prostate, colorectal, and ovarian) is shown as well in Table 1. Not only are the number of aberrant cells enriched higher for the leukemia and multiple myeloma diseases compared to epithelial cancers, but the number of non-diseased cells enriched is higher as well compared to epithelial cancers due to the fact that non-diseased blood cells can carry the same antigens as CLCs and CPCs, whereas for CTCs the blood cells do not express EpCAM. Due to the higher number of cells anticipated for the leukemic and multiple myeloma diseases, we had to build devices with high numbers of microtraps.

### 3.2. Microfluidic Containment Device Operation 

**Figure 2:**
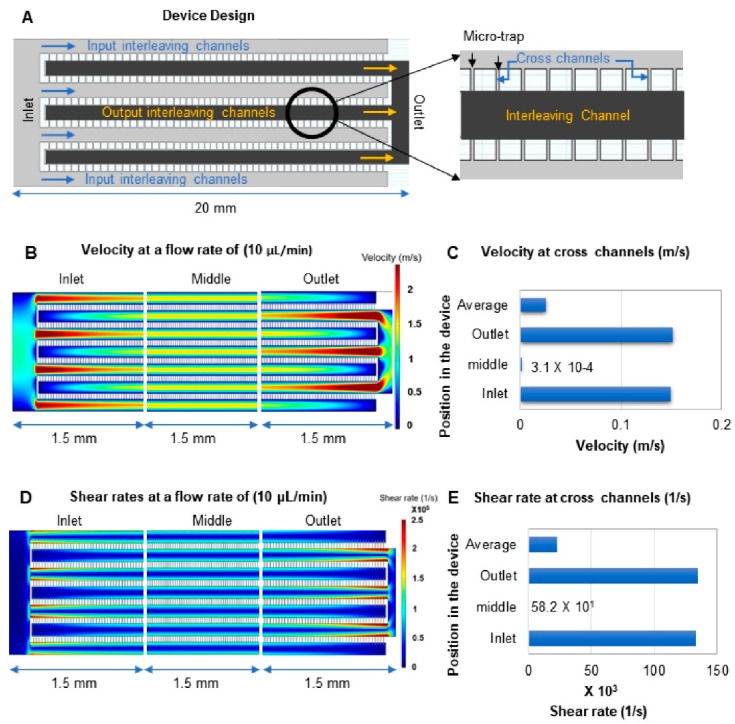
Simulations of the microtrap device. (**A**) 2-D CAD design of the microtrap device used for COMSOL simulations showing the interleaving network for the flow of fluid, and the cross-channels, which produce the microtraps when a cover plate is sealed to the device. The magnified image of the microtrap area is shown on the right with a single interleaving output channel (red) and two interleaving input channels (gray). (**B**) The simulated linear fluid velocity throughout the microtrap chip. The simulation shows three sections of the device: (i) input section; (ii) middle section; and (iii) outlet section. Flow was simulated across the interleaving input/output channels and the cross-channels. The dashed box shown here is the region of the device that was simulated in Figure S3 (see Supplementary Materials). (**C**) Bar graph representing the mean velocities expressed in m/s observed for the cross-channels at different sections of the device and at a 10 μL/min volume flow rate. The sections labeled here correspond to the sections of the device simulated in (**B**). (**D**) Simulated shear rate at three different sections of the device, inlet, middle, and outlet sections. (**E**) Bar graphs representing the mean shear rates across the cross-channels at different sections of the device at a volume flow rate of 10 μL/min. The sections of the device listed here correspond to those sections shown in (**D**).

The microtrap device (both single and 8-bed devices) used two independent networks of interleaving channels that were interconnected using smaller cross-channels positioned orthogonally to the interleaving channels. The cross-channel, when sealed with a cover plate, generated a pore structure whose dimensions were determined by the size of the cross-channels (Figure 2A) and allowed fluid flow between the input and output interleaving channels. During operation, CPCs/CLCs are released from the isolation chip and directed to the microtrap device, where they become physically trapped with the efficiency of trapping dependent on the size of the microtrap with respect to the cell of interest. Trapped cells could then be immunophenotyped or subjected to FISH followed by imaging with a fluorescence microscope to read out the appropriate signals. Microtrap beds were connected in parallel to provide sufficient numbers of equally accessible pores to retain a large number of cells (see Table 1). If the eight beds were connected in series, a majority, if not all, of the cells would be retained within the first bed, generating cell pileup and crowding, thus making imaging difficult. Conversely, placing the beds in parallel (see Figure 1A) allowed access to all beds from a common input, and thus, dispersed the cells with equal probability at the 80,000 containment pores associated with the 8-bed device. 

Even though earlier studies from our group showed that live cells with a cell diameter of ~16 µm (CTCs) were successfully contained by microtraps with dimensions of 8 × 4 µm^2^ [31], the current study required containment of smaller cells and cell numbers that were higher (see Table 1). RPMI-8226 cells (multiple myeloma model) have a size range of 6–16 µm with an average diameter of ~13 µm whereas SUP-B15 cells (B-ALL model cell line) have an average size of ~10 µm with a size range of 8–12 µm. Thus, when devices with microtraps of 8 × 4 µm^2^ (width × depth) were used, we observed that the containment efficiency was <50% (data not shown). However, devices with microtraps of 4 × 2 µm^2^ produced a containment efficiency >90% for these cells (Figures S1 and S3B). 

### 3.3. Device Design and COMSOL Simulations

Finite element analysis (COMSOL) was performed on the microtrap device to deduce the projected linear velocity through the fluidic network and the corresponding shear rates to help determine the containment efficiency of the microfluidic device to physically trap live cells without damaging them. Laminar flow was validated across the entire device for the following flow rates: 1, 3, 5, and 10 μL/min with an aqueous fluid (Figure S3). The rationale behind choosing these flow rates was to test the optimal flow rate for effective containment of live cells without damage but having sufficient pressure to fill the device without generating air bubbles. The cells used for our studies were human cancer cell lines having a diffusion coefficient of 5 × 10^−14^ m^2^/s [36]. 

**Figure 3:**
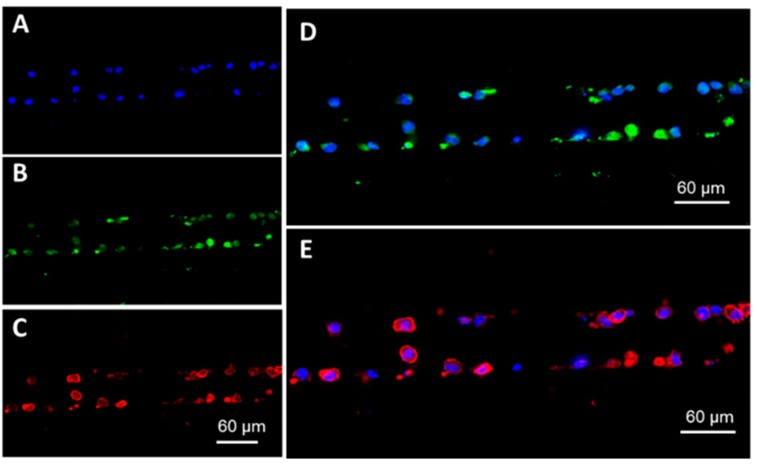
On-chip immunophenotyping of RPMI-8226 cells. (**A**) DAPI-labeled RPMI-8226 cell nucleus aligned at the entrance of the microtraps formed by the cross-channels and the cover plate assembled to the device. (**B**) CD138 expression of the RPMI-8226 cells and (**C**) CD38 expression for the same cells. (**D**) Composite image of CD138 expression (FITC channel) with the cell nucleus (DAPI channel of the microscope). (**E**) Composite image of CD38 expression (APC channel) with the cell nucleus that was DAPI stained. Exposure times were DAPI 50 ms, FITC 500 ms, and APC 1500 ms with 20× magnification. All images were collected using the Keyence fluorescence microscope. Shown in this fluorescence image are cells aligned along one interleaving input channel with cross-channels on either side of that channel.

**Figure 4:**
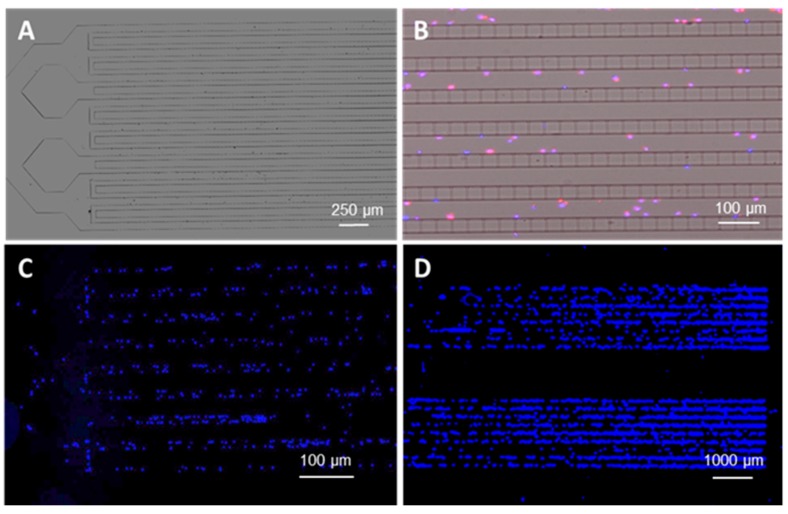
(**A**) Brightfield image of the bifurcated entrance channels of the microtrap device. RPMI-8226 cells were injected into the device at 10 μL/min and contained at the entrance of the microtraps. Cell images were processed according to the procedure listed in the materials and methods section of this manuscript and labeled with DAPI (nuclear stain) and CD38-APC markers. (**B**) Brightfield image merged with DAPI and APC channels showing the presence of the cell nucleus and CD38 on the cell surface aligned mainly at the microtrap entrances. (**C**) Entrance of the single bed device imaged using DAPI. RPMI-8226 cells were trapped inside the device at the entrance to the microtraps. (**D**) Two consecutive beds of the 8-bed device imaged with the DAPI channel of the microscope for stained RPMI-8226 cells.

The flow within the microtrap device was driven hydrodynamically, hence, a parabolic flow profile existed with higher velocity in the center of each individual channel as compared to the channel walls (no-slip condition; see Figure 2B) [37]. Figure 2A shows a CAD drawing of the device with 4-µm-wide microtraps and a depth of 2 µm. This device was found to provide 90% containment efficiency for unfixed RPMI-8226 cells as shown in Figure S3A. For larger epithelial cells (i.e., SKBR3), the containment efficiency was 96%. Cells were evenly distributed throughout the microtrap device as well (Figure 3; Figure 4). In the experiments evaluating trapping efficiency, the cells were DAPI stained and counted using a microscope to verify the number of cells captured. Cells that were not retained were collected at the outlet of the microtrap device into a flat-bottomed plate and enumerated and inspected for damage. The containment efficiency was determined from the ratio of cells in the microtrap to the total number of cells introduced (i.e., cells trapped and cells passing through the microtrap device).

The average velocity at the inlet and outlet interleaving channels toward the input end of the device was 2.5 m/s and in the interleaving channels in the center of the device it was 1.5 m/s at a volumetric flow rate of 10 µL/min. Even at these relatively high velocities, the flow was still laminar (see Figure S3B,C). Accounting for differences in the velocity between the interleaving and cross channels, the average velocity was calculated for the cross-channels at different sections of the device at a 10 µL/min volume flow rate and is plotted in Figure 2C.

The average linear velocity in the cross-channels was 0.02 m/s at the 10 µL/min volumetric flow rate. To measure the pressure drop across the device, the relative pressure at the outlet was defined in absolute terms (*p_A_ = p + p_ref_*, where *p_A_* is the absolute pressure, *p* is the relative pressure, and *p_ref_* is the reference pressure, which was set to 1 atm (101 kPa) [3]. A gradual drop in pressure across the length of the device was noted, with this drop being ~14 kPa (16 and 2 kPa at the inlet and outlet, respectively, at 10 µL/min). The calculated shear rates at different volumetric flow rates were used to determine the shear stress in the microtrap device [38]. According to Newton’s law, shear stress is the shear rate times the viscosity:

Shear stress (dynes/(cm^2^)) = Shear rate (1/s) × T, (1)

where T is the dynamic viscosity (T for water is 8.90 × 10^−3^ dynes*s/cm^2^ at 25 °C).

We calculated the average shear stress on the cells experienced in the microtrap device through the entire device at different flow rates. At a flowrate of 1 µL/min, the shear rate calculated was 6042 s^−1^, which corresponds to a shear stress of 54 dynes/cm^2^ and is 10 times higher at 10 µL/min (Table 2). Moreover, higher shear rates were observed in the inlet and outlet of the device, where cells have potentially the highest probability of being damaged when flowing near the wall of the device as opposed to the center of the channel or the center area of the device where lower shear stress is observed (Figure 2D,E). Shear rate distributions across a section of the device can be found in Figure S3D,E.

The shear stress experienced by cells in physiological conditions as they travel through capillaries and arterioles ranges between 40 and 55 dynes/cm^2^ [39,40,41,42]. Interestingly, even though the shear stress at a flow rate of 10 μL/min was 10 times higher than the average shear stress of cells traveling through arterioles, we did not observe obvious damage of RPMI-8226 or SUP-B15 cells when contained at the entrance of any microtrap within the device. Cells tolerate transiently high shear stress of ~3000 dyn/cm^2^ or higher [43,44]. In our microtrap device, the average transit time of the cell traveling through the chip is 210 ms if it is not retained by the microtrap and only 1 min when cells are retained within the microtrap device. Because the cells experience high shear stress briefly, they remain intact during the transport in high shear stress environments [45].

As a control for on-chip immunophenotyping using the microtrap device, we performed immunophenotyping on a slide (see Figures S4 and S5) to compare with our on-chip results. RPMI-8226 cells were labeled with FITC-anti-CD138 antibodies and APC-anti-CD38 antibodies. RPMI-8226 cells express these markers [3,46,47]. See Figures S4 and S5 for the flow cytometry results for RPMI-8226 and on-slide immunophenotyping data, respectively.

Figure 3A shows DAPI-stained cells positioned at the microtraps in the single bed device. Here, we injected live cells followed by introduction of a fixative (2% PFA) to demonstrate the ability to fix cells on-chip. It was evident that our device could contain live cells without damaging their integrity. Trapped cells were stained with anti-CD138-FITC and anti-CD38-APC human antibodies (Figure 3B,C, respectively). Figure 3D,E show the composite images of the cell nucleus (DAPI channel) with the corresponding FITC and APC fluorescence emission signals.

Imaging of retained cells in the device for immunophenotyping was rapid. The microtrap device could be imaged at 20× magnification for all three colors (DAPI 50 ms, FITC 500 ms and APC 1500 ms) in <2 min. An advantage of using the microtrap device is the fact that single cells are positioned at the micropore entrance in a 2–D format, making them easy to locate. For immunophenotyping using the 8-bed device, >98% of cells were imaged in one plane without requiring z-stacking using a 20× microscope objective.

### 3.4. Microchip Processing and Imaging of a Large Number of Single Cells

Results for isolating CTCs, CLCs, and CPCs are summarized in Table 1, and show a high number of cells enriched from 1 mL of a patient blood sample using the sinusoidal microfluidic enrichment chip when analyzing CPCs and CLCs due to the fact that the enrichment antibody also selects non-diseased cells as opposed to CTCs, where the enriched fraction possesses only a few non-diseased cells. As such, thousands of cells may be required to be analyzed via immunophenotyping or FISH [3] to identify cancer cells (i.e., CLCs and CPCs). To facilitate the analysis of a vast number of cells, we designed the microtrap device with eight beds capable of entrapping enriched cells, subject them to staining, and present them in a 2-D array format for microscopic evaluation.

The 8-bed microtrap device possessed 80,000 containment pores patterned in PDMS from SU-8 reliefs. Three-level reliefs (i.e., three different heights of microstructures; see Supplementary Materials for fabrication description) were required to reduce the pressure in the chip and achieve well-balanced flow through the entire fluidic network (Figure 1 and Figure 3). Because CLCs and CPCs have a diameter ranging from 6–16 µm (see Table 1), to ensure maximum containment efficiency by the microtraps, we used 4 × 2 µm^2^ cross-sections for the containment microtraps (see Figure S3A). Fluorescence microscope images of cells contained at the microtraps and immunostained are presented in Figure 4.

Figure 4A shows a brightfield image of a single bed in the 8-bed device. Merged images of cells aligned at micropore entrances were imaged with DAPI (50 ms acquisition time) and anti-CD38-APC antibodies (1500 ms acquisition time) to identify CD38 on the RPMI-8226 cell surfaces; see Figure 4B. Figure 4C shows a single bed device containing DAPI-stained cells. DAPI-stained cells in two consecutive beds from the 8-bed device are imaged and presented in Figure 4D. As can be seen from Figure 4C,D, the contained cells are fairly well distributed throughout the microtrap 2-D array in spite of the decrease in the linear velocity seen down the length of the interleaving input channels (see Figure 2B). We noticed no loss of cell integrity at the microtraps even in the region of the input/output ends of the microtrap array where the shear stress was high (Figure 3D). Finally, when the flow was stopped, the cells remained at their trapped location.

### 3.5. On-Chip FISH

**Figure 5:**
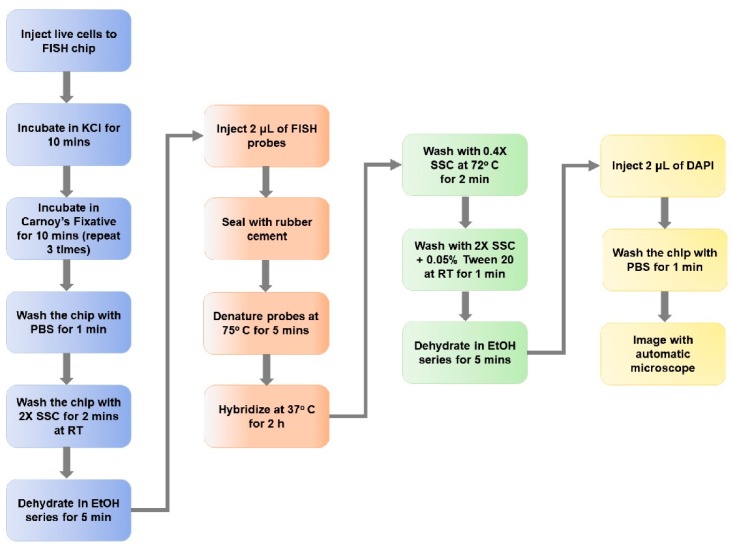
Workflow of FISH using the microtrap device. The workload was reduced from 2 days (slide method) to 4 h using the microtrap device primarily due to the hybridization time reduced from overnight to 2 h. The probe volume required for the assay was also reduced from 10 to 2 μL as well as using the microtrap device. Live cells were injected into the microtrap device at a flow rate of 10 μL/min and the washing steps were done at 5 μL/min to reduce the shear stress on the fixed cells contained within the microfluidic device.

The microtrap device can be used for immunophenotyping and cytogenetic analysis, such as FISH. FISH determines aberrations in a metaphase chromosome or chromosomes buried in interphase nuclei from a fixed cytogenetic sample. The procedure for FISH processing on-chip is detailed in the materials and methods section as well as the Supplementary Materials. FISH experiments were carried out on RPMI-8226 and SUP-B15 cells with Cytocell FISH probes. The conventional workflow using microscope slides for FISH is a tedious and time-consuming process (see Figure S6), which requires 2–3 days including overnight hybridization of FISH probes. Figure 5 shows a step-by-step workflow for the on-chip FISH procedure, which required ~4 h of processing time and 2 µL of stock FISH probes for the 8-bed microtrap device, producing a 5-fold reduction in FISH probe volume compared to the slide-based FISH assay.

**Figure 6:**
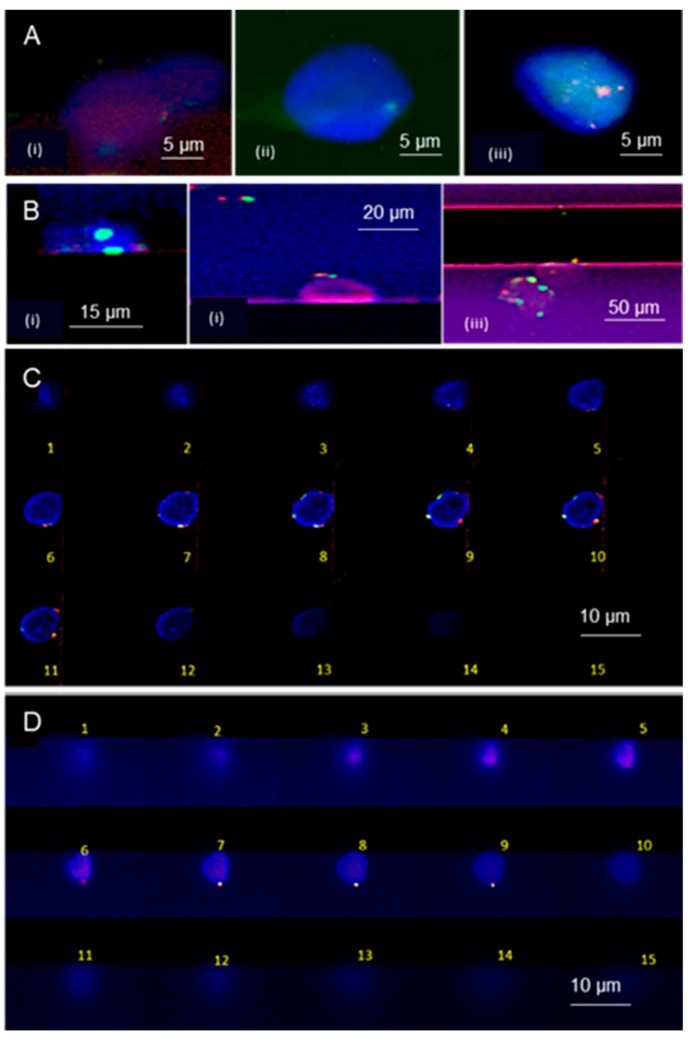
FISH-on-chip analysis of RPMI-8226 and SUP-B15 cells. (**A**) RPMI-8226 cells after FISH processing using the microtrap chip with the D13S319 plus deletion probe; (i) a cell that shows one green and one red FISH signal; (ii) a cell with only one green signal; (iii) a cell with 2 green and 2 red signals. (**B**) FISH analysis of SUP-B15 cells processed with the TEL/AML1 translocation, dual fusion probes showing the TEL (ETV6, 12p13.2) region in red, and AML1 (RUNX1, 21q22.12) region in green. (i) Two cells contained at the entrance of two different microtraps, but the FISH probes were visible in only one cell with two green signals; (ii) shows one red and two green signals with no clear yellow signals present; (iii) two cells that show distinct red and green signals, one cell captured at the entrance of microtrap shows one red, and one green signal with a possible yellow fusion signal. Both (**A**,**B**) were imaged in one single z-plane without z-stacking. (**C**,**D**) show z-stacking planes of 15 different image planes for FISH images from SUP-B15 cells captured at the microtrap and FISH processed with BCR/ABL plus translocation, dual fusion probe. (**C**) SUP-B15 cell with two green and two red signals. (**D**) SUP-B15 cell with one yellow fusion signal (second yellow signal not visible) and one red and green signal. Each image shows 15 separate images through the 15 μm distance range taken at 1-μm imaging intervals. FISH probes were specific to the BCR/ABL gene region, Philadelphia (Ph) chromosome tagging. All images were acquired using a Nikon 60× oil objective with DAPI—200 ms, FITC—1500 ms, TRITC—2500 ms integration times. The average SNR was 59 for the green probe and 68 for the red probe.

Figure 6A (i–iv) shows FISH signals from RPMI 8226-cells processed on-chip. FISH signals present in both the red and green channels of the fluorescence microscope in all of the cells were seen except for the image shown in Figure 6A (ii), where only one green signal was present due to deletion of the target gene region. In some of the cells, only one set (1 red and 1 green) of signal was present. One reason for losing some FISH signals is that the cells possess a 3-D structure (Figure 1E) even after entrapment at the microtrap and the fact that a high numerical aperture (NA) microscope was used with a short focal length; the images shown in Figure 6A were processed using only a single imaging plane (*z*-axis). This issue was addressed by using z-stacking of the imaging planes over a range equal to the average cell diameter.

Figure 6B shows a set of SUP-B15 cells processed using the microtrap chip for the TEL/AML1 FISH probes imaged in one image plane (i.e., no z-stacking). For TEL/AML1, probe TEL (ETV6—Erythroblastosis Variant Gene 6 translocation, ETS) refers to a region in chromosome 12 p-arm (12p13.2), and AML1 (or RUNX1—Runt-Related Transcription Factor 1) refers to the region in the q-arm of chromosome 21 (21q22.12). In a normal cell, there should be two red and two green signals and in a diseased cell, two yellow fusion signals are expected due to translocation of the TEL and AML1 genes. All of the images (Figure 6B) showed distinct red and green signals, with no clear indication of a yellow fusion signal to identify any cell as positive for the t(12;21) translocation.

Figure 6C,D shows two examples of SUP-B15 cells processed with BCR/ABL1 FISH probes for Ph t(9;22) (q34.12; q11.23) imaged with z-stacking (1-µm increments along the *z*-axis over ~15 µm). Ph t(9;22) (q34.12; q11.23) consists of two gene regions, with one from chromosome 9 corresponding to ABL1 gene (red labeled) and the other for the BCR (breakpoint cluster region) gene in chromosome 22 (green labeled). In a cell without a chromosomal fusion aberration, there are two green and two red signals. If there are yellow fusion signals detected, the cell can be identified as Ph(+). Imaging of 1-µm z-planes over a 15-µm range covered the entire cell as noted in Figure 6C,D. Those images showed two or more signals present within the cells entrapped by the microtrap device. Figure 6C shows two distinct red and green signals (confirmation as a cell not possessing the fusion product) and Figure 6D shows one yellow fusion signal (second signal not visible or merged with the first one) and one red and green signal, confirming it as a B-ALL cell that is Ph(+).

### 3.6. Measurement of MRD Status in Pediatric B-ALL Patients Using CLCs

**Figure 7:**
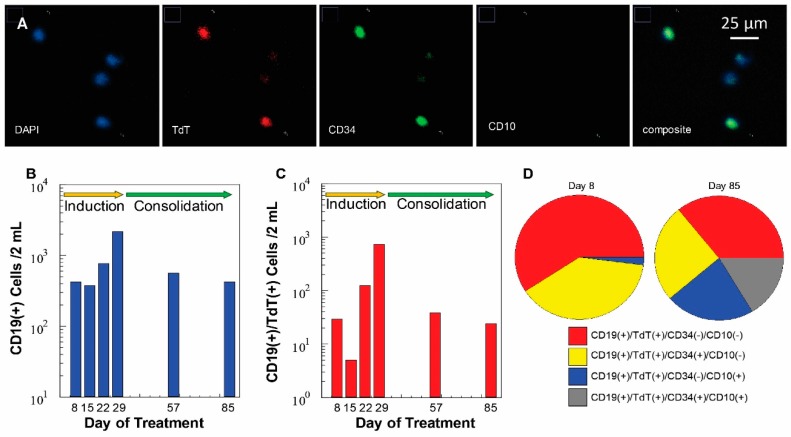
(**A**) Immunophenotyping of cells enriched from peripheral blood of a B-ALL patient by targeting cells with that express the CD19 antigen. The cells were stained using DAPI (nucleus), and monoclonal antibodies directed against TdT (FITC), CD34 (Cy3), and CD10 (Cy5). The images were acquired using a 40× microscope objective. The CLCs shown were DAPI(+)/CD34(+) and TdT(+), but CD10(−). (**B**) Microfluidic monitoring of a B-ALL patient from day 8 to 85 of chemotherapy. Total cell count represents all DAPI(+)/CD19(+) cells selected. (**C**) Number of CLCs identified as DAPI(+)/CD19(+)/TdT(+)/CD34(±)/CD10(±). (**D**) Change in phenotype among CLCs for this patient for days 8 and 85.

CLCs and normal B-cells were affinity selected from a patient’s peripheral blood with anti-human CD19 antibodies attached to the surface of a CLC enrichment (sinusoidal) microchip via a single-stranded oligonucleotide cleavable linker containing a dU residue [34]. Blood was collected from a pediatric patient (1–18 years) diagnosed with B-ALL undergoing induction and consolidation chemotherapy. Released cells following enrichment were immunophenotyped to distinguish CLCs from normal B-cells using the microtrap device; cells that demonstrated the expression of terminal deoxynucleotidyl transferase (TdT) in the nucleus were classified as CLCs. Additionally, the staining cocktail contained CD19/CD34/CD10 fluorescently labeled monoclonal antibodies to provide additional phenotypic data (Figure 7A).

We analyzed the blood of a pediatric B-ALL patient to determine MRD status during chemotherapy on days 8, 15, 22, 29, 57, and 85 (Figure 7B–D). The clinical specificity was determined based on a threshold value established from the analysis of healthy donors as negative controls (average CD19 expressing cells was 68 cells/mL of peripheral blood). Grounded on that, we classified this patient as MRD(−) upon completion of induction and consolidation therapy on day 85. In this particular patient, on day 85, we observed a new phenotypic population of cells (Figure 7D) not observed during the first two analyses, which were secured on days 8 and 15 during induction therapy. Cells with the CD19(+)/TdT(+)/CD34(+)/CD10(+) phenotype began to appear in the blood on day 22. Although leukemic cell phenotype changes are common in B-ALL due to the effects of steroids as part of chemotherapy (i.e., loss of CD34), it is likely that the aforementioned cells represent normal immature lymphoid precursors whose morphology and immunophenotype are similar to the CLCs found in B-ALL. MRD status of this patient was determined to be positive only once, which was on day 29 of treatment when the level of enriched cells classified as CLCs (361 cells/mL) were above the threshold value.

**Figure 8:**
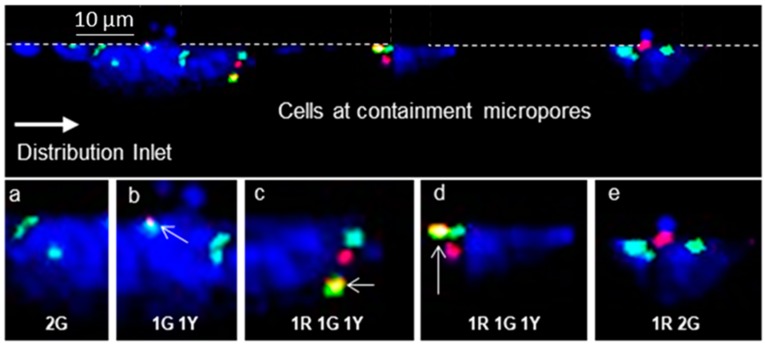
On-chip FISH processed B-cells isolated from a diagnosed B-ALL patient. TEL/AML1 FISH probes were used for the chromosomal aberration of t (12;21) translocation. Cells were imaged at the microtraps. Zoomed images show (**a**) single cell with 2 green FISH signals; (**b**) single cell with one green and one yellow signal; (**c**) a single cell with one red, one green, and one yellow FISH signal; (**d**) single cell with one red, one green, and one yellow FISH signals close to each other in the cell; and (**e**) single cell with one red and two green signals. In all cases, the images were collected using a 60× objective with z-stacking.

To confirm the chromosomal status of the CD19-expressing cells on day 29 of treatment, we tested the enriched cells for chromosomal aberrations via FISH. Figure 8 shows a stitched image of cells contained on the microtrap device from a B-ALL patient. The sample was processed using TEL/AML1 FISH probes, which were able to identify the t(12;21) translocation. The TEL/AML1 fusion FISH probes identifies the most common rearrangements in childhood B-ALL, which is seen in around 17% of patients [48]. Figure 8a shows a single cell with two green FISH signals and Figure 8b shows two green FISH signals, with one signal showing a yellow signal (see arrow). Figure 8c shows a single cell with discrete red, green, and yellow signals. There was no visible evidence of a second yellow signal for confirmation of both t(12;21) translocations. Figure 8d shows a cell with the same observation of one red, one green, and one yellow signal, which were closely packed together. In Figure 8e, there are two green and one red signal visible. Figure 8a,b,e shows a lack of a red signal from the TEL gene. It has been observed that in B-ALL patients, there is the possibility for deletion of one TEL allele [49,50,51].

## 4. Discussion

FISH testing constitutes important and independent prognostic factors and is considered obligatory for analyzing patient outcome [52]. Of the current ~117 human genetic tests approved by the US Food and Drug Administration, 18 of these are FISH-based assays and most are directed toward hematological diseases, such as AML, multiple myeloma, and ALL.

AML arises from mutations occurring in progenitor cells of the myeloid lineage, which results in the inability of these cells to differentiate into functional blood cells. AML is the most common adult leukemia, with >21,000 new cases in the US in 2018, with a 5-year survival rate of 25%. The primary cause of death for AML patients is due to disease relapse [53]. The WHO currently categorizes patients into four groups [54]. For example, one category is patients with recurrent genetic abnormalities, which can consist of seven different chromosomal aberrations (typically balanced translocations or inversions, inv). Some of these aberrations are t(8;21)(q22;q22) associated with the *RUNX1*/*RUNX1T1* genes in chromosome 8, inv(16)(p13.1q22)/t(16;16)(p13.1;q22) occurring in the *CBFB*/*MYH11* genes of chromosome 16, and inv(3)(q21q26)/t(3;3)(q21;q26) of the *RPN1*/*EVI1* genes in chromosome 3. While AML MRD is typically managed using bone marrow biopsies, we have shown that CLCs can be used to determine recurrence from MRD in AML. The CLCs were enriched from blood samples using three sinusoidal microfluidic devices, with each one targeting a specific AML-associated antigen, CD117, CD34, and CD33 [54].

Multiple myeloma is associated with the abnormal expansion of terminally differentiated B clonal plasma cells in the bone marrow that produces an abnormal monoclonal paraprotein [55,56]. Multiple myeloma has three clinically defined stages: (i) MGUS (monoclonal gammopathy of undetermined significance), which is the asymptomatic stage; (ii) SMM (smoldering multiple myeloma) an intermediate phase; and (iii) the symptomatic stage referred to as active multiple myeloma [57]. In most cases, bone marrow biopsies are used to manage multiple myeloma. However, we and others have shown that CPCs can be used to manage this disease, which used a minimally invasive liquid biopsy [3,4,31]. In our study, we used a microfluidic device containing an array of sinusoidal microchannels with anti-CD138 monoclonal antibodies used to enrich CPCs from multiple myeloma patients [3]. It has been reported that in 16–50% of all multiple myeloma cases, chromosome 13q aberrations are present [58,59]. More than 90% of reported cases show the chromosomal aberration specifically in the 13q14 region [60]. We were able to perform FISH in the CPCs to detect the presence of chromosome 13q deletions using a slide-based FISH method (see Figures S6 and S7).

The FISH probes used for the RPMI-8226 cells, a model of multiple myeloma, identifies the DLEU region of chromosome 13 covering the 13q14 gene and used a red (APC channel) fluorescent probe. The control gene, 13qter located at the end of chromosome 13, was labeled with a green fluorescent probe (FITC channel). In a normal cell, there are two green signals and two red signals. However, due to the polyploidy nature in some cells, there may be multiple chromosomes (>2). In CPCs, it is expected that one or both DLEU regions (DLEU1 and DLEU2) may be deleted [61].

Figure 3 and Figure 6 show immunophenotyping and FISH processing of RPMI-8226 cells using our microtrap device. As expected, the data seen in Figures S4 and S5 in the Supplementary Materials and our previous studies [3] confirmed the expression of CD138 and CD38 proteins for RPMI-8226 cells. We detected the presence of chromosome 13 as a green FISH signal corresponding to the 13qter gene (100 kb), which was present in all images, as shown in Figure 6A. Figure 6A (i) and (ii) shows deletion of the red signal corresponding to gene regions covering DLEU1, DLEU2, D13S319, D13S272, and RH47934 (156 kb) as expected for the RPMI-8226 cell line, as well. Most of the RPMI-8226 cells contained both red and green signals (Figure S7 in Supplementary Materials) lacking deletion, which is consistent with the karyotype data for this cell line.

B-ALL is the most common cancer diagnosed in children, representing ~30% of cancer diagnoses [62]. Despite significant improvements in the overall survival of children with B-ALL, there is a group of patients that experience relapse and ultimately die from their disease [63]. In fact, the likelihood of relapse is 80% for patients who have MRD at the end of induction therapy, indicative of active disease [64]. Monitoring of MRD, therefore, is considered a powerful predictor of outcome in B-ALL.

Cytogenetic abnormalities detected at diagnosis or generated during chemotherapy constitute important prognostic factors [52]. In B-ALL patients, 25–30% of patients have hyperdiploidy, 25% have t(12;21), 3–5% have t(9;22), 10% have MLL translocations, and 2% have iAMP21 chromosomal abnormalities. Once an aberration is detected, it can aid in the determination of the treatment regimen [65,66]. As another example, the detection of specific chromosome aberrations, such as t(9;22)(q34;q11.2) for *BCR-ABL1*, which results in the formation of the Philadelphia (Ph) chromosome, or t(12;21) aberrations of TEL/AML1 gene translocations are used to assign B-ALL patients to specific targeted therapies [67].

For the SUP-B15 cell line, which is a model for B-ALL [68], there are a few targeted gene variations that are typically evaluated using FISH [50,69,70]. MLL break-apart probes are used to detect the breakage of the MLL gene, which is frequently found in infant B-ALL [71,72,73]. BCR/ABL1 probes are used to detect the formation of the Philadelphia (Ph) chromosome produced by the fusion of two genes from chromosome 9 and 22, which is one the most important prognostic indicators for several hematological disorders, including B-ALL (see Figures S7, S8, and S9 for on-slide FISH analysis for some of these chromosomal abnormalities) [74,75,76].

In this study, the SUP-B15 cells were tested for TEL/AML1 translocations and BCR/ABL1 (Ph chromosome) using the microtrap device for FISH. For the SUP-B15 cell line, it is expected to see two distinct red and two green signals with the TEL/AML1 FISH probes. The TEL (ETV6) gene region marked in red corresponds to the 12p13.2 in chromosome 12 covering 168 kb of D12S1898 region and the green marker covers AML1 (RUNX1) in chromosome 21q22.12, a 167 kb gene region, including the CLIC6 gene [50]. On-chip FISH results (Figure 6B) showed distinct red and green signals corresponding to the presence of chromosome 12 and 21. Lack of a yellow signal confirmed t(12;21)(p13.2;q22.12) translocations were not present in the SUP-B15 cell line, which agrees with the karyotype as noted in the literature. The Philadelphia chromosome results from translocations of the ABL1 gene (9q34.11-q34.12, red) in chromosome 9 and the BCR gene (22q11.22-q11.23, green) in chromosome 22. The BCR probe region covers the GNAZ and RAB36 genes in a 169 kb region plus 148 kb region in telemetric BCR. The ABL1 probe covers a 346 kb region in the middle of the FUBP3 gene. As for the karyotype data of SUP-B15, we expected to see >90% of cells possessing the Ph chromosome (yellow fusion signal present). No yellow signal would be considered a cell with no Ph chromosome.

Figure 6C showed that SUP-B15 cells processed on-chip with BCR/ABL1 genes expressed two distinct red and green signals. This confirmed that there was no Ph chromosome present while in Figure 6D it showed the presence of one yellow fusion signal, confirming Ph(+) in that cell. In Figure 6D, we did not identify the fusion signal in this cell. However, the patient sample processed for FISH on-chip showed improved FISH signals as seen in Figure 8.

The microtrap FISH assay resulted in an SNR of 59 for the green signal, and 68 for the red signal. In the case of the slide-based assays, the SNR for the green and red signals were 64 and 63, respectively (see Figure S9), indicating that the ability to detect single molecules associated with the fluorescent reporter attached to each FISH probe was clearly visible using the microtrap device. The challenge is that in the FISH experiments, we used a high numerical objective with a small focal distance and as seen in Figure 1E, z-stacking was necessary to cover the genetic material housed within the nucleus. This may have been the reason that some signals were missed. This can be obviated by using a high numerical objective with a larger focal distance to better cover the entire nuclear region when the cells are located at the pore entrance.

Most FISH-based assays are predicated on the use of bone marrow, which is enriched in diseased cells compared to blood. For example, in the case of multiple myeloma, CPCs in peripheral blood are reported to be >100-fold lower than in bone marrow [77,78]. If disease relapse and chromosomal defects could be detected from peripheral blood, painful bone marrow biopsies could be avoided, and physicians could obtain information in near real time and potentially implement changes in treatment to affect better outcomes for patients with hematological diseases.

To obviate the need for a bone marrow biopsy, we used a liquid biopsy secured from a B-ALL patient using an affinity microfluidic chip and performed immunophenotyping and FISH on those enriched cells using our microtrap device. Similar to our previous work on the isolation of leukemic cells from blood of patients diagnosed with AML, a sinusoidal microfluidic chip with positive affinity selection was used [3,4], but in this case the affinity selection used a different antibody (anti-CD19 monoclonal antibodies) to enrich B-cells. While the enrichment of the CLCs and CPCs in our previous work was accomplished using a microfluidic chip, the downstream analysis was done off-chip, including immunophenotyping and FISH. Standard FISH workflow demands highly trained and experienced personnel (see Figure S6), making it difficult to implement in clinical laboratories not possessing the specialized facilities and trained personnel. Additionally, blood cells collected from a bone marrow biopsy are used for cytogenetic analysis, requiring an invasive procedure [64].

Using our microfluidic assay, a blood sample was subjected to affinity enrichment with high efficiency in terms of recovery and purity, and thus, the entire leukocyte population of peripheral blood did not require cytogenetic interrogation. The only cell population interrogated was those that expressed the target antigen, which in the case of B-ALL was CD19-expressing cells. Additionally, enriched cells were distributed in an array-like format as determined by the position of the microtraps of the microtrap device (see Figure 4C,D), which made them easier to image as opposed to stochastically distributed on a properly functionalized surface to induce cell adhesion to the surface. In addition, the 8-bed version of the microtrap device possessed 80,000 pores for retaining cells. While the data displayed in Table 1 show the total number of cells affinity selected (aberrant and non-aberrant) were 5314 for AML, 2840 for ALL, and 7775 for multiple myeloma, these numbers were based on a per mL sample volume. In some cases, it may be necessary to use larger input volumes, such as 10 mL, to search for rare CLCs and CPCs to find cells in the correct phase to elicit proper FISH signals. In these cases, the full advantage of the large dynamic range of the 8-bed device can be realized.

To reduce the workflow for FISH, microfluidics has been suggested by several groups, with the processing time reduced from several days using conventional slide-based FISH to several hours using FISH-on-chip platforms [14,26,27,28]. In addition, microfluidics has also resulted in a reduction in the use of expensive FISH probes. However, the reported platforms (see Table S1) required the use of special surface coatings to allow for cells to adhere to the surface of the device. Because of the stochastic nature of the attachment to the surface of the chip, this can create cell aggregates that made it difficult to image single cells under high magnification to determine the chromosomal status of the cells. Our device obviated the need of surface coatings and ordered the cells in a 2-D format to reduce device preparation steps and simplify single cell imaging, respectively. The microfluidic was comprised of an array of microtraps that were easily formed via a replication step in PDMS from a relief prepared by lithography. The relief also possessed the fluidic network.

We showed in this work that the microtrap device could be coupled to a rare cell enrichment chip to allow processing of circulating cells, such as CPCs or CLCs, with the ability to perform immunophenotyping and FISH of the enriched cells directly from blood samples. Moreover, experiments showed that our microtrap device was capable of containing live cells with >90% efficiency with sufficient traps to process CLCs and CPCs enriched from blood. The microtrap device was operated at a 10 µL/min volume flow rate to facilitate proper filling of the device without air bubbles. At this flow rate, even though cells experienced ~570 dynes/cm^2^ shear stress, no obvious cell damage was observed for cells contained by the microtraps. Cell physical survival was attributed to the cell membrane’s ability to handle relatively high shear stress for a brief time.

Using our 8-bed device with 80,000 microtraps, we could process thousands of cells for molecular profiling following enrichment. FISH results were achieved in <4 h by reducing the hybridization time from overnight to 2 h. Also, automated imaging was demonstrated for phenotyping in 2 min and FISH imaging in < 5 min, reducing the workflow for FISH compared to conventional slide-based assays, which requires 2–3 days of processing time, most of which is done manually. The FISH-on-chip provides full process automation.

When the cells were physically retained at the microtraps, they did possess a 3-D structure, requiring z-stacking to cover all of the FISH probes present inside the cell nucleus. Unlike immunophenotyping, where it was possible to use a single focusing plane because a lower magnification and associated longer focal length was required, FISH required the use of a high numerical aperture objective with a smaller focal length to capture high resolution images along several focal planes to image all of the FISH probes present in the cell nucleus. Even though z-stacking was necessary for FISH imaging, it was possible to automate this process. We set a common upper and lower threshold point in the device and selected the points where the cells were present at the arrayed microtraps and proceeded with automated imaging with pre-set exposure times for different filters (DAPI 200 ms, FITC 1500 ms, Cy3/Cy5 2500 ms). Processing of the captured images using BZ-X Analyzer and FIJI is detailed in the Supplementary Materials.

As opposed to our previous version of this device, which possessed larger microtraps [31], this device was designed to accommodate smaller CLCs and CPCs and the higher number of cells to analyze (see Table 1). For example, the CLCs (i.e., B-ALL cells) are smaller than CTCs and, as such, required the use of a smaller containment pore (4 × 2 µm^2^ compared to 8 × 6 µm^2^ in our previous report) [31]. Because the affinity selection process for CLCs and CPCs results in the enrichment of a much larger number of cells due to the fact that the even non-diseased cells express the capture antigen, a larger number of containment pores were required (80,000 herein compared to 5000 in our previous device) [31]. In addition, our previous report only performed immunophenotyping and did not carry out FISH on the chip, as was demonstrated here.

In the current rendition, the microtrap device was made from PDMS by casting it against a relief. However, the same device architecture can be made from a thermoplastic, such as cyclic olefin copolymer (COC), which has some decisive advantages compared to PDMS [79]. For example, because COC is a thermoplastic, it can be injection molded to allow production of devices at high rates and at significantly lower chip cost compared to PDMS [79]. In fact, the entire device can be injection molded in a single cycle, with the only requirement being cover plate bonding as a finishing step. Also, COC has excellent optical properties that allow for high sensitivity imaging using the spectral range typically employed for FISH [27]. COC can be UV/O_3_ activated to change its wettability to allow efficient filling with aqueous solutions without creating air bubbles and does not show the typical rapid hydrophobic recovery as seen with PDMS [80]. This will allow for the generation of a low-cost disposable appropriate for in vitro diagnostics. When the microtrap device is physically integrated to the cell enrichment device via a fluidic motherboard, fully automated processing of liquid biopsy samples can be envisioned to enable clinical use.

## 5. Conclusions

The ability of our microtrap device was demonstrated using CPCs and CLCs for immunophenotyping and FISH analyses of relatively small cells (D_avg_ ~12 µm) and in high numbers. The same device could be used to identify expression patterns of proteins and detect targeted chromosomal aberrations in single cells. Using the 8-bed device with 80,000 containment microtraps, we could process thousands of live or fixed cells enriched using a cell isolation microchip. FISH results were achieved in <4 h by primarily reducing the hybridization time from overnight to 2 h, and lowering the volume of the FISH probes required for analysis. The microtrap device was used for automated imaging for phenotypic identification of cells in 2 min and for FISH in <5 min for all fluorescence channels without the need to scan a relatively large area. Moreover, we were able to enrich B-cells from an ALL patient and process those cells to identify chromosomal aberrations. In future work, the use of a thermoplastic, such as COC, instead of PDMS will be undertaken to produce a low-cost, disposable device appropriate for use in clinical applications.

## Figures and Tables

**Table 1:** Number of CLCs and CPCs compared to CTCs detected in cancer patients.

Cancer Cells (Antigen Used for Selection)	Target Cells (mL^−1^)	Non-Aberrant Cells (mL^−1^)	Cell Diameter (µm)	Reference
CLCs (AML, CD33, CD34, CD117)	11–2684	10–2450	11–16	[4]
CLCs (B-ALL, CD19)	40–840	400–2050	6–12	this work
CPCs (CD138)	10–5900	43–1875	12–16	[3]
CTCs in epithelial tumors (EpCAM)	1–800	3–10	10–23	[35]

Note: The references used here were taken from a single type of cell selection chip (sinusoidal device) so that comparisons could be made as to the numbers of the CLCs, CPCs, and CTCs secured from liquid biopsies.

**Table 2:** Average shear rate and calculated shear stress on cells at each microtrap for the flow rates listed.

Flow Rate (μL/min)	Shear Rate (1/s)	Shear Stress (dynes/cm^2^)
1	6042	53.8
3	18,206	162.0
5	30,454	271.0
10	63,750	567.4

## Supplementary Materials

The following are available online at https://www.mdpi.com/2073-4409/9/2/519/s1, Table S1. Summary of FISH-based microfluidic technologies. Figure S1: metrology of microtrap device, Figure S2: fabrication steps for 8-bed microtrap device, Figure S3: COMSOL simulations of microtrap device, Figure S4: flow cytometry results of RPMI-8226 cells, Figure S5: immunophenotyping of RPMI-8226 cells, Figure S6: chart of FISH workflow using slide-based method, Figure S7: slide-based FISH results for RPMI-8226 cells, Figure S8: slide-based FISH results for SUP-B15 cells, Figure S9: slide-based FISH results for SUB-B15 cells.
