# Supplementary material for: Microfluidic Device for On-Chip Immunophenotyping and Cytogenetic Analysis of Rare Biological Cells
Source: Cells. 2020 Feb 24;9(2):519. doi: 10.3390/cells9020519 (PMC7072755; doi:10.3390/cells9020519)
Supplement: Supplementary file 1 [file cells-09-00519-s001.pdf]

Supporting Information

Microfluidic Device for On-Chip Immunophenotyping and Cytogenetic Analysis of Rare Biological Cells

K. M. Weerakoon-Ratnayake[1,2], S. Vaidyanathan[2,3], N. Larky[2,4], K. Dathathreya[1,2], M. Hu[2,4], J. Jose[2,7], S. Mog[1,2], K. August[5], A. Goodwin[4], M.L. Hupert[6*], M.A. Witek[1,2*] and S. A. Soper[1,2,4,6.7*]

[1]*Department of Chemistry, The University of Kansas, Lawrence, KS, USA*
[2]*Center of Biomodular Multiscale Systems for Precision Medicine, Lawrence, KS, USA*
[3]*Bioengineering, The University of Kansas, Lawrence, KS, USA*
[4]*University of Kansas Medical Center, Kansas City, KS, USA*
[5]*Children's Mercy Hospital, Kansas City, MO, USA*
[6]*Biofluidica Inc., BioFluidica Research Laboratory, Lawrence, KS, USA*
[7]*Department of Mechanical Engineering, The University of Kansas, Lawrence, KS, USA*

Chemicals and reagents. FISH probes were purchased from Cytocell (Cytocell Ltd, New York, NY,USA), and cell surface markers from BioLegend (San Diego, CA, USA). Saline-sodium citrate (SSC) and colcemid were obtained from Fisher Scientific, DAPI (4′,6-diamidino-2-phenylindole) counterstain (1 µg/mL) and counterstain diluents were received from Kreatech Diagnostics (Leica Biosystems Inc. IL, USA). Purified monoclonal anti-CD138 (Clone #359103, 0.5 mg/mL) antibody was received from R&D Systems (Minneapolis, MN). PBS buffer and trypsin were purchased from Sigma-Aldrich as well as bovine serum albumin (BSA; in PBS buffer, pH 7.4). For immunofluorescence, antibodies such as anti-CD138 FITC (MI15, 5.0 µg/mL), anti-CD45-FITC (HI30 clone, 10 µg/mL), anti-CD56-PE (MEM-188 clone, 20 µg/mL), anti-CD38-APC (HIT2 clone, 2.5 µg/mL) were purchased from BioLegend and prepared in 100 µL of PBS. All solutions were prepared in nuclease-free water (Invitrogen, Carlsbad, CA).

Cell culture. The SUP-B15 (ATCC® CRL1929™) cell line was purchased from American Type Culture Collection (ATCC, Manassas, VA) and was grown in 20% fetal bovine serum (FBS) and 80% Iscove's modified Dulbecco's medium with 4 mM L-glutamine adjusted to contain 1.5 g/L sodium bicarbonate and supplemented with 0.05 mM mercaptoethanol. The RPMI-8226 (ATCC® CCL155™) cell line was also purchased from American Type Culture Collection and cultured in RPMI 1640 with 2.5 mM L-glutamine supplemented with 10% FBS (GIBCO, Grand Island, NY). The cell lines were incubated at 37˚C under a 5% CO_2 atmosphere. For sub-culturing, cells were grown as non-adherent cell suspensions in T25 culture flasks (Corning) by maintaining a cell density between 5 x 10^5 and 2 x 10^6 cells/mL with fresh media changes every 2-3 d either via dilution or replacement of new medium.

Fabrication of microtrap device. The single bed microtrap device consisted of 2 levels; one level with an interleaving channel network and the second level with cross channels that formed the microtraps to retain biological cells for FISH or immunophenotyping. The interleaving channels were ~30 µm deep and the second level contained the cross-channels that were either 4 or 2 µm deep. SU-8 lithography was performed using two optical masks with the appropriate alignment marks to allow for two-step photolithography. SU-8 structures were formed on a Si wafer that contained metal-patterned alignment marks. The microtraps, which were formed by the cross channels when a cover plate was bonded to the substrate, consisted of a 4 or 2 µm thick layer of SU-8 2002 spin coated at 3000 rpm for 30 s and soft baked at 100˚C for 3 min. The wafer was then exposed to 160 MJ/cm^2 of UV light for 28 s through a dark field glass mask using a Quintel exposure station with the mask alignment marks aligned to the optical mask. Post exposure baking was done at 100°C for 4 min followed by SU-8 development.

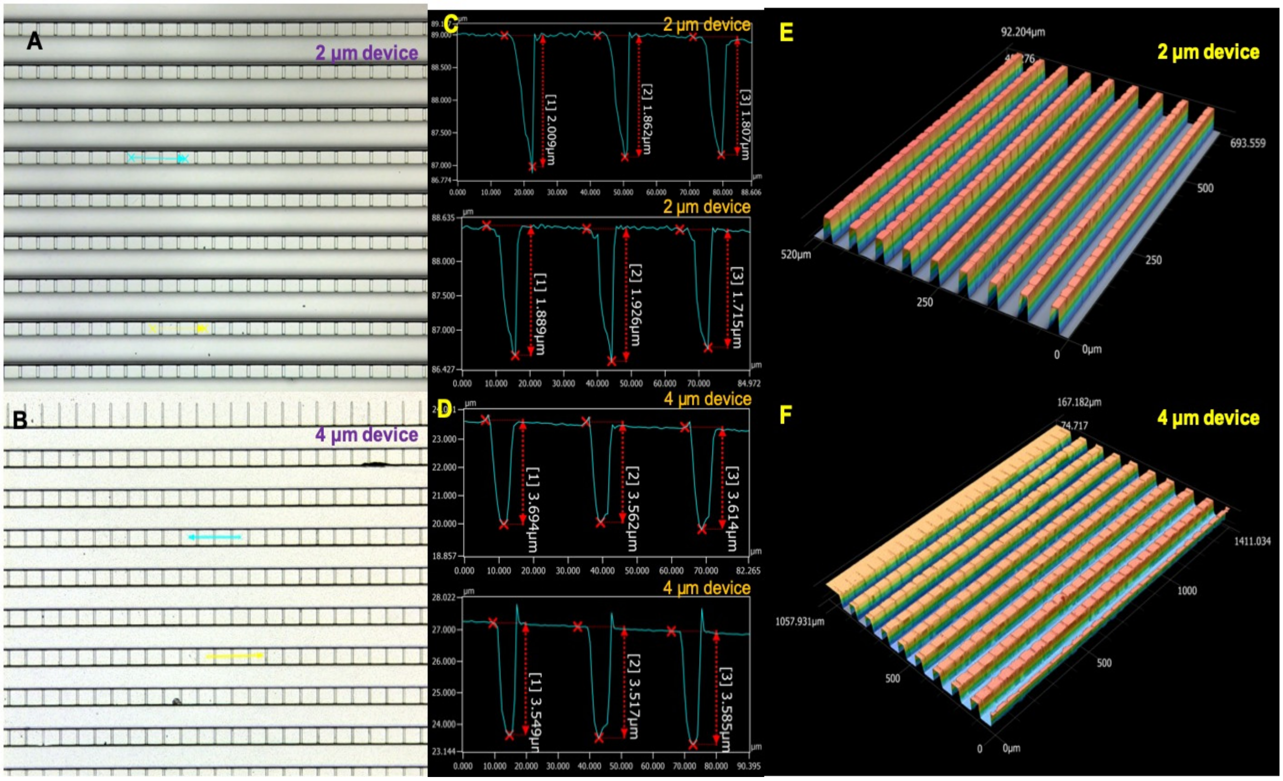

Figure S1. Microscopic brightfield image of the microtrap device with microtraps containing: (**A**) Two µm depth; and (**B**) four µm depth. Depth profile for yellow and light blue lines show 10 distinct line scans performed to obtain the average depth of the microtraps. Profile analysis of the microtraps shows, (**C**) 2-µm deep microtraps measured to be 1.514 ±0.094 µm; and (**D**) 4-µm deep microtraps showing an average of 3.524 ±0.123 µm. Laser 3-D profile of the microtrap device for: (**E**) 2 µm depth; and (**F**) 4 µm depth. Blue area shows a depth of ~30 µm and the light red area shows the surface where the microtraps are located. All data were secured uisng a Keyence VK-X200 series laser scanning confocal microscope for 3-D and profile measurements. The dimensions of the trapping region were 40 mm in length and 25 mm in width.

For the interleaving channels, which consisted of input/output channels that were interconnected using the cross-channels, SU-8 2015 was spin coated at 1300 rpm for 30 s to yield a 30 μm thick resist layer on top of the exposed cross-channel layer. Soft baking was done at 100°C for 10 min. Fiducial (alignment) marks were used for proper alignment of the second mask with respect to the exposed wafer. UV exposure was performed at 300 mJ/cm^2 for 53 s and post baked at 100°C for 10 min. The wafer was developed first in SU-8 developer for 5 min followed by fresh SU-8 developer solution for 2 min and rinsed with IPA (isopropyl alcohol) for 30 s. After drying, the wafer was hard baked at 150°C for 20 min and 70°C for 10 min to anneal surface cracks in the resist, which were evident after development. The developed wafer was then cleaned using a plasma asher at 600 W for 2 min and used as a relief for subsequent PDMS casting.

The SU-8 relief consisted of different designs with different pore widths (4 μm, 6 μm or 8 μm; not shown here). Figure S1A and B shows an example of a bright-field image of the microtrap device transferred into PDMS where line scans are shown in Figures S1C and S1D for 4 μm and 2 μm microtrap devices (depth of traps), respectively. A 3-D scan of the device is shown in Figure S1E and F, where the blue area represents the ~30 μm deep interleaving channels and the microtraps are shown in the light red area. Measurements of the depth of the microtraps were analyzed using the Keyence VK-X200 series laser scanning confocal microscope. According to the measurements, 4 μm deep microtraps showed an average of 3.5 ± 0.1 μm from depth analysis of the PDMS device while 2 μm deep microtraps showed a depth of 1.5 ± 0.1 μm, where both gave ~0.5 μm depth deviations from the designed value.

Figure S2 shows the fabrication steps for the 8-bed microtrap device. Similar to the single bed device, it was fabricated on a Si wafer using SU-8, but in this case it required 3 lithography steps. As with the single bed device, the Si wafer possessed alignment marks for the 3 lithography steps. Lithography Step 1 was performed to fabricate the microtrap layer by spin coating SU-8 with a controlled thickness of 2 or 4 μm. Photoresist was exposed through a mask to define the microdtrap positions and following SU-8 development, the cross-channels were generated. Next, SU-8 photoresist was spin coated onto the Si wafer followed by UV exposure through another mask and resist development to fabricate the interleaving channels (lithography Step 2). The SU-8 layer thickness for the interleaving channels were ~30 μm in depth. The final lithography step (Step 3) utilized an SU-8 layer (~150 μm) processed as described for Steps 1 and 2, but with a different mask to fabricate the distribution channels that possessed 150 μm depths. The distribution channels were used to allow for one input port that supplied sample to all 8 beds with a common output port. The fluid flow into the distribution channels was perpendicular to the fluid flow in the interleaving input channel network to assure uniform fluid supply to all 8 beds.

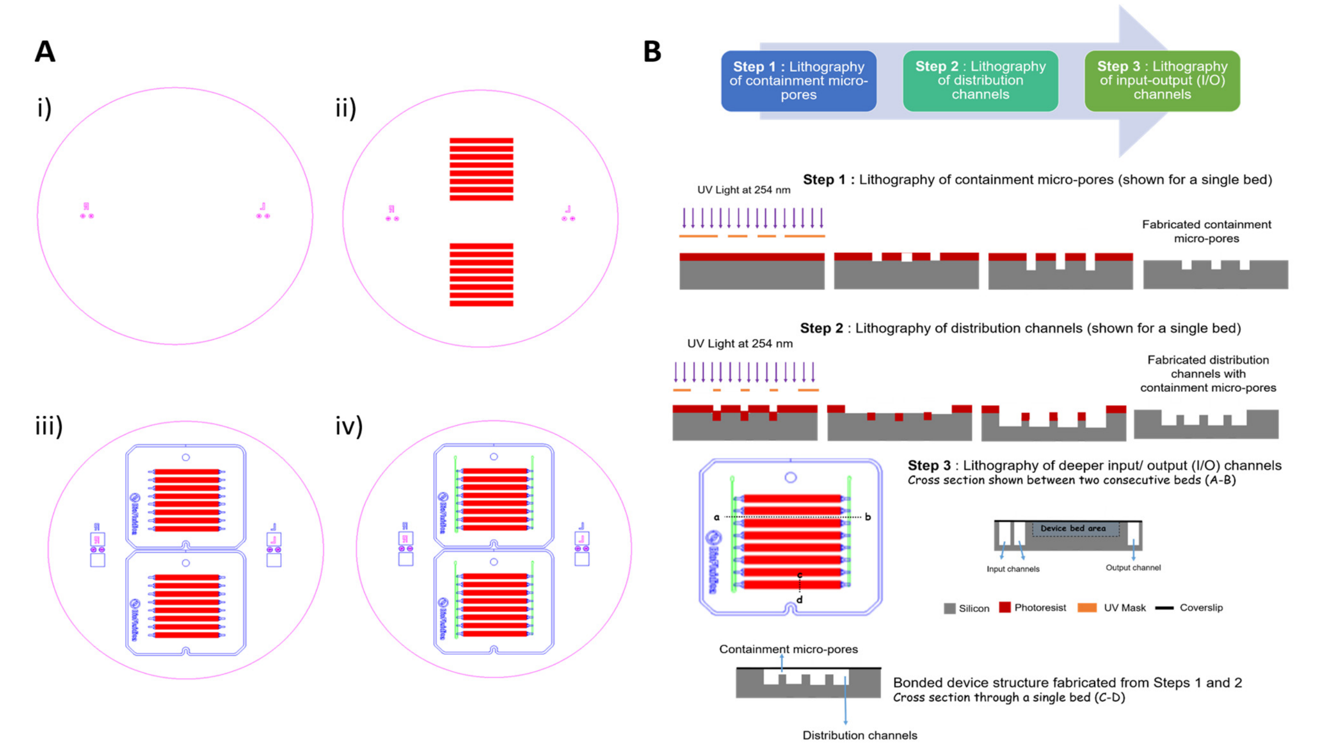

Figure S2. (A) (i) Fabrication of alignment marks; Cr deposition and Au layer deposition on a Si wafer followed by spin-coating photoresist, UV exposure, and development of the resist to produce the alignment/fiducial marks. Afterwards, resist and Cr were stripped leaving only the alignment marks on Si and the next photolithography steps were performed. (ii) Fabrication of microtrap layer of 2 μm thickness. (iii) Fabrication of interleaving channels with 30 μm resist thickness. (iv) Fabrication of distribution channel layer with 150 μm thickness. **(B)** The overall process of 3-step lithography on the Si wafer. After the alignment marks were fabricated as shown in (**A**), Step 1 was performed to fabricate the microtraps. SU-8 was spin coated onto the Si wafer and microtrapping structures (2 or 4 μm depth) were produced by exposing the resist to UV light followed by development and etching. Step 2 was performed on the Si wafer repeating the same processes as in Step 1, but with a different mask. Here, the depth of the interleaving channels were designed to be ~30 μm. Step 3, which is the final step, was performed to fabricate the deeper distribution channels with a depth around 150 μm.

Flow simulation of the microtrap device. COMSOL simulations were performed to determine the proper fluidic operational parameters for containing biological cells using the microtrap device without causing damage to the cells so that they could be subjected to immunophenotyping or FISH. For the simulation, we chose the device design with microtraps of 4 μm width and 2 μm depth as they provided the highest containment efficiency (see Figure S3A).

Figure S3B shows the linear velocity profile through a small section of the device for a 10 μL/min input volumetric flow rate (2x magnified view). The region simulated was 3.5 mm from the entrance side of the device with a simulated length of 0.8 mm (total length of the trapping region is 16 mm; see Figure 3 in the main text for the region of the device simulated here). The profile line graph associated with the linear velocity across a series of interleaving channels (see yellow line in Figure S3B) at 10 μL/min is shown in Figure S3C. The linear flow velocity in the center of the interleaving channels was higher (~2.75 m/s; average) compared to the cross-channels forming the microtraps, where the velocity was ~0.5 m/s (average). The highest velocity reached inside the device at 10 μL/min volumetric flow rate was ~3 m/s

in the area where the fluid enters the input interleaving channels. As can be seen from Figure S3B, the input interleaving channels seems to show a rather constant linear velocity down the length of this channel network, but does decrease further down the device as noted in Figure 3, which is a consequence of the fact that these channels have a termination point. The output interleaving channels show the reverse behavior because the input side of these channels possesses the termination point.

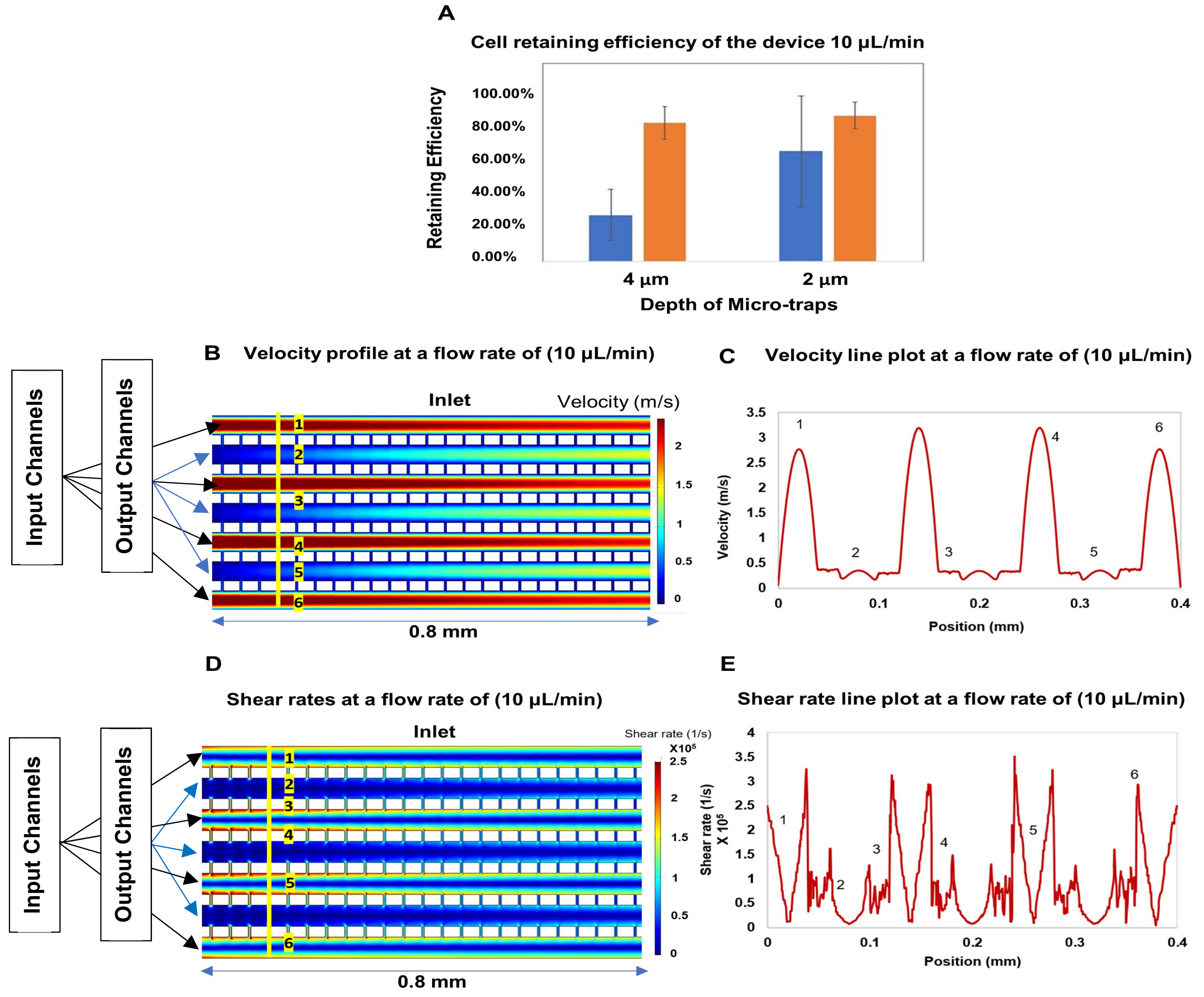

Figure S3. (**A**) Optimized containment efficiency of RPMI-8226 cells using 4 µm or 2 µm microtrapping devices, where the dimension represents the depth of the cross-channels (widths were 4 µm in both cases). A flow rate of 10 µL/min was used for evaluating the containment efficiency of these devices. Overall, devices with 2 µm depth showed the highest containment efficiency. (**B**) Magnified image of the velocity profile across a section of the microtrap device showing the velocity in the interleaving channels and in the cross-channels. The velocity profile of the device is represented by the yellow line and further explained in the line graph. (**C**) Line graph showing different velocities for different sections of the device. (**D**) Shear rate through a section of the device. The walls and the microtrap region have maximum shear rate where the probability of the cells experiencing disruption is high. Shear stress was predicted using Newton's law. (**E**) Line graph representing a profile of the device's distributed shear rate. The center of the channels has much less shear rate due to the lack of friction from the walls compared to the microtraps, which exert higher shear on the cells. The region simulated in (**B**) and (**D**) is noted in Figure 2B of the main text with a dashed box. Note: input and output interleaving channels are labeled.

The velocity at all of the input interleaving channels near the walls and in the middle area of these channels were 5 X 10^{-4} m/s and 1.75 m/s, respectively, as determined by COMSOL – this is consistent with the fact that at these flow rates, the flow is laminar with the velocity higher at the center of each channel and lower at the walls due to the no-slip condition (this was seen for all channels of the device, including the cross-channels). The line graph shows the flow profile at the entrance of the device where the velocity was higher (represented by positions 1, 4 and 6) at the center of the interleaving input channels and the velocity being lower at the interleaving output channels (represented by positions 2 and 5) as well as the cross-channels (position 3). This relationship is only seen at the input side of the device as indicated by the yellow line shown in Figure S3C.

Figure S3D shows the shear stress in the input and output interleaving channels as well as the cross-channels as calculated using Newton's law (in this case, the same section of the device was simulated as shown in Figure S3B and C). The line graph in Figure S3E shows the shear rates at positions of the device as designated by 1-6 listed in Figure S3D. The interleaving channels had lower shear rate in the middle of the channel. Higher shear rates were found in close proximity of the walls (*i.e.*, 10 µm), consistent with the results shown in Figure S3B and C. In the microtraps, higher shear stress was observed compared to the input/output interleaving channels. However, it is important to note that cells do not travel through the microtraps, see Figure S3A. Similar to the velocity, the shear rates decreased in both the input interleaving channels and the cross channels down the length of the device. Table 2 in the main text shows the shear stress values corresponding to the average shear rates across the device at each volumetric flow rate.

Fabrication of the enrichment microfluidic device. Detailed information regarding the fabrication of the enrichment chips, monoclonal antibody attachment using cleavable oligonucleotide linkers, and protocols for cell isolation and release have been provided in our previous publications [1, 2]. Briefly, enrichment chips were fabricated in cyclic olefin copolymer, COC (TOPAS 6013S-04, TOPAS Advanced Polymers) using hot embossing from brass molds. Each chip possessed an array of 50 sinusoidal microfluidic channels with the geometry optimized for high cell recovery and purity. Each microchannel was 25 µm wide, 150 µm deep, and 30 mm long. The input channel of the chip was attached to capillary tubing to allow for connection to a syringe pump to allow for sample and wash buffer introduction. Microfluidic channels were UV/O_3 activated for 13 min at 22 mW/cm^2 using a commercial UVO Lamp (Model 18, Jelight Company), which produced surface-bound carboxylic acids. Following photochemical activation, chips were flooded with a solution of 40 µM ssDNA cleavable linker in a 20 mg/mL EDC in PBS buffer (pH 7.4) and incubated at room temperature for 2 h or overnight at 4°C to covalently attach the ssDNA linker at its 5'-terminus to the activated COC surface. After the reaction was complete, the microfluidic chip was rinsed with 200 µL of PBS (pH 7.4). This was followed by infusing into the chip 300

mM DTT solution in carbonate buffer (pH 10) for 20 min to reduce the 3'-disulfide group into a reactive sulfhydryl moiety (-S-H). The microfluidic chip was quickly rinsed with 100 µL of PBS (pH 7.4) and immediately flooded with an aliquot of an antibody modified with SMCC (0.8 mg/mL) in PBS. The reaction proceeded for 2 h on ice or overnight at 4°C. Antibody modification was as follows: 1 mg/mL antibody in water was modified with SMCC (50x molar excess) for 30 min. Antibody-SMCC was purified on a ZEBA desalting column (7k MWCO) according to the manufacturer's protocol.

Flow cytometry of RPMI-8226 cells. All flow cytometry studies were performed using a BD Accuri C6 Plus instrument (BD Biosciences, San Jose, CA). The flow cytometer was equipped with 2 lasers consisting of a blue (488 nm) and red laser (640 nm) with four photodetectors. The software used for data collection and analysis was the BD Accuri C6 Plus software. For sample preparation, approximately 10^6 RPMI-8226 cells obtained from culture media were centrifuged and resuspended in 1 mL cold 0.5% BSA/PBS buffer. Prior to staining, the cells' surfaces were blocked with human IgG and incubated at 4°C. Ten-µL of 0.1 mg/mL fluorescently-labeled antibody or isotype control was added to the cell suspension and left to incubate for 45 min at 4°C under dark conditions. Upon completion of the antibody reaction, cells were washed 3 times by centrifuging and replacing the buffer with cold 0.5% BSA/PBS.

On-slide immunophenotyping. Cultured cell lines were aliquoted to 500-1,000 total cells in 250 µL and transferred into a cytospin funnel locked in a cytospin fixture and mounted with a poly-lysine coated glass slide. These funnels were then cytospun at 1100 g for 7 min. Following centrifugation of cells onto the poly-lysine slide, cells were fixed to the slide with 2% PFA (100 µL) followed by 3 washing steps with 100 µL of PBS. Slides were placed into a Sequenza cover plate for immunostaining in a Sequenza slide rack (ThermoFisher). Mixtures of the immunostaining markers were prepared in PBS buffer in a total volume of 150 µL for each slide with the required concentrations of the immunostains. The solution was applied to the slides and allowed to flow through the liquid surface interface. Slides were incubated at room temperature for 40 min followed by 2 washing steps with PBS buffer (1000 µL). Next, DAPI II in 0.1% Triton-X was introduced and incubated for 10 min. Slides were then washed with water and PBS. Finally, slides were covered by a cover glass (No.1) and sealed with nail polish prior to imaging.

Conventional FISH. Conventional FISH protocols were adapted from Kreatech and Cytocell. For control samples, RPMI-8226 or SUP-B15 cells in media were spun down and the supernatant was replaced with KCl hypotonic solution (0.056 M). After 10 min of incubation, an equal volume of fixative (3:1 (v/v) methanol:acetic acid) and cell suspension was added and the cells were spun down again. Ice cold Carnoy's fixative was added dropwise dissolving the resulting cell-pellet, which was fixed for 10 min. The aforementioned procedure was repeated 3 times. The cells were then stored in methanol:acetic acid at 20°C until required for FISH.

Cells were re-suspended in freshly prepared Carnoy's solution before used in FISH analyses. Cells were spotted onto a glass slide and the slides were immediately placed on a hot plate at 37°C and left to dry for ~15 min increasing the temperature to 80°C. The slides were treated with 70% acetic acid for 30 min and 2X SSC (pH 7.3) at 37°C (±1°C) for 5 min followed by dehydrating successively in an ethanol series (70%, 85%, and 100%) at room temperature for 2 min each and allowed to dry completely as pre-denaturation steps. Ten µL from the stock FISH probe solution was heated to 37°C (± 1°C). The FISH probe mixture (10 µL) was applied to each slide and covered with a cover slip carefully followed by sealing with rubber cement and allowed to dry completely. Cells with probes were denatured at 75°C (±1°C) for 7 min and hybridized at 37°C (±1°C) overnight in a Bambino oven (Boekel Scientific Bambino II™ Hybridization Oven). The next day, all of the post-hybridization steps were performed.

After overnight hybridization, the cover slip was removed. Slides were then washed with by 2X SCC/0.05% Tween-20 (wash buffer II) at room temperature for 2 min then with 0.4X SSC/0.3% NP-40 (wash buffer I) at 72°C (± 1°C) for 2 min and again with wash buffer I for 2 min at room temperature without agitation. Slides were washed with ethanol for 2 min and air-dried. Then, 10 µL (0.1 ng/mL) of DAPI II counterstain (Kreatech) was applied to each slide. The cells were imaged using a Keyence BZ-X710 microscope equipped with a Nikon CFI Plan Apochromat *VC* 60x oil immersion objective lens, NA 1.4, WD 0.13 mm, F.O.V. 25 mm, DIC, Spring Loaded (Nikon Inc., Melville, NY, USA) and the images were analyzed and processed with the Keyence BZ-X Analyzer and FIJI software.

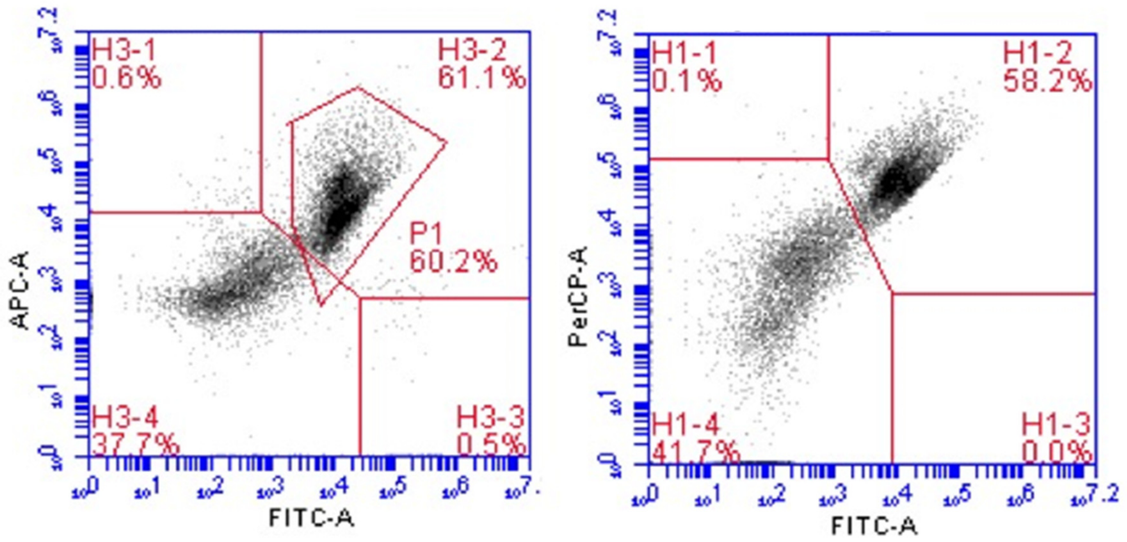

Figure S4. Fluorescence scatter plots for surface antigen expression of the RPMI-8226 cell line. For the left–panel, the APC-A channel is measuring fluorescence from CD56 and the FITC-A channel is monitoring fluorescence from CD138. For the right panel, the PerCP-A channel is measuring fluorescence from CD38 while the FITC channel is used to monitor fluorescence from CD56.

Phenotypic characterization of RPMI-8226 cells via flow cytometry. CPCs primarily express CD38, CD138, and in a few cases CD56. Additionally, expression of CD45 is very weak or not expressed at all. Figure S4 provides flow cytometry scatter plots showing the expression patterns of CD56, CD38 and CD138 for RPMI-8226 cells, which serve as a model for CPCs associated with multiple myeloma. From the plot, 98% of the cells expressed CD38, 90% expressed CD138, and 74% expressed CD56 with <0.001% expressing CD45, consistent with literature data for this cell line [3, 4].

On-slide immunophenotyping of the RPMI-8226 cell line was also used to further determine protein expression. This CPC model cell line expressed CD138 (FITC channel in Figure S5A(i)), CD38 (APC channel in Figure S5A(ii)), and CD56 (APC channel in Figure S5B(i)), but did not express CD45 (FITC channel in Figure S5B(ii)).

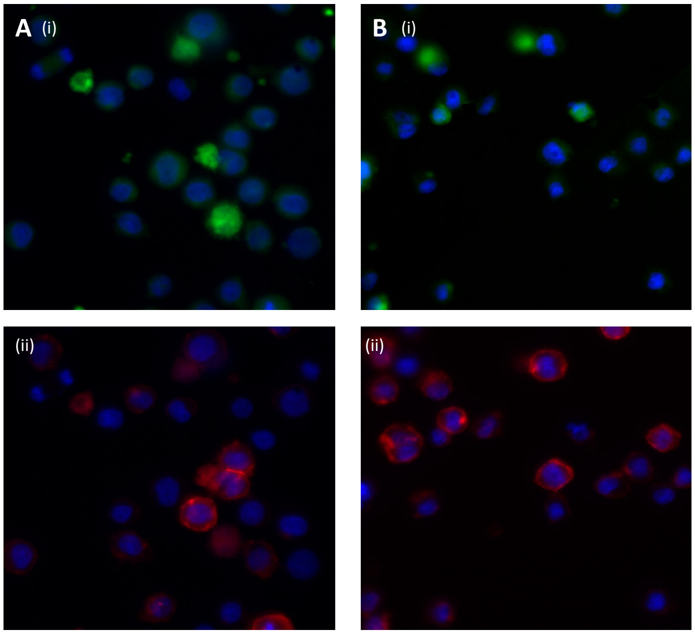

Figure S5. On-slide immunophenotyping of RPMI-8226 cells. (**A**) (i) CD138 in FITC channel and (ii) CD38 in the APC channel. (**B**) (i) CD56 in FITC channel and (ii) CD45 in the APC channel. All images were combined with fluorescence data from the DAPI channel, which is a nuclear staining dye. Exposure times were DAPI 50 ms, FITC 1500 ms and Cy5 3000 ms.

On-slide (conventional) FISH. Figure S6 shows the FISH procedure carried out on-slide as a control to validate our on-chip FISH processing and imaging. The slide preparation is represented as a schematic shown in Figure S7A for a general overview of the process. Figure S7B shows FISH processed cells (RPMI-8226) that were deposited onto a glass slide and imaged by a fluorescence microscope within a cytogenetic laboratory. The images were acquired using a Nikon 60x oil immersion objective. Image

processing was accomplished using a Keyence BZ-X Analyzer and FIJI software as mentioned in the experimental section. Figures S7B, and C show the presence of 13qter, and 13q34 green fluorescent labeled telomeric region to identify chromosome 13. If the cells contain both red signals with two green signals, it represents cells that do not contain a chromosome 13 deletion. Some cells showed only one or no red signal, which indicated a chromosome 13 deletion.

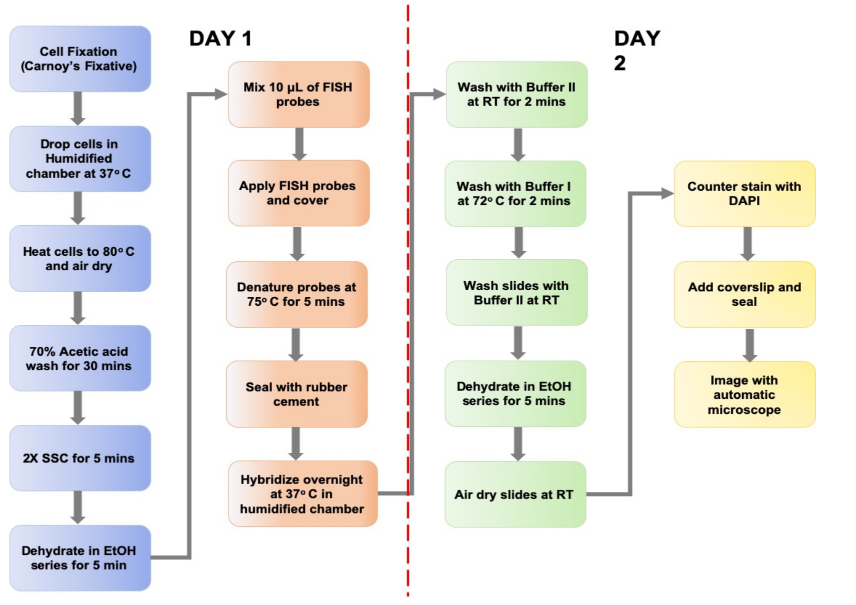

Figure S6. Workflow for FISH process using the conventional slide-based protocol. Basically, the process required a 2-day workflow with overnight hybridization.

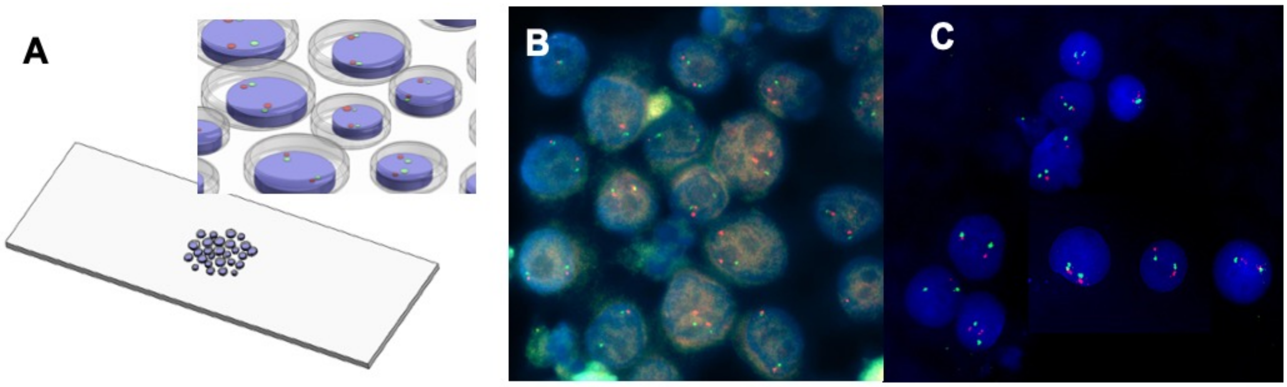

Figure S7. FISH analysis of RPMI-8226 cells on (**A**-**C**) microscope slides. (**A**) Schematic showing cells deposited onto the microscope slide with a slight flattening of the cells. (**B**) Fluorescence image obtained using a 100x oil immersion objective and dedicated FISH microscope at a Cytogenetic Laboratory. (**C**) Fluorescence images of FISH processed RPMI-8226 cells on-slide acquired using a Keyence microscope equipped with a Nikon 60x oil objective with DAPI, FITC (green), and Cy5 (red) filters.

Automated imaging system for FISH. For immunophenotyping, one can image with wide-field microscopy and use a lower magnification objective, such as a 10X objective. Using our microtrap device, each bed can be imaged completely in 3-5 min at 60X magnification with sufficient data accumulation time for detecting FISH probes. For FISH experiments reported herein, slides were imaged using multi-point analysis and z-stacking in all color channels after loading the FISH processed cells onto the

microscope stage. Low magnification was used to spot the cells with FISH signal that could be further analyzed using a higher magnification objective. These points were then marked in the xy plane with multi-point imaging. Upper and lower thresholds were set to image all of the FISH probes contained within the nucleus of the cell and images were captured using automated imaging via a Keyence BZ-X700 microscope in several z-planes.

Figures S8 and S9 show composite images from DAPI, FITC (green) and Cy5 (red) color channels. The objective used was a Nikon CFI Plan APO *VC* 60X (oil) and N/A 1.4 with a working distance of 0.17 mm. Images were taken as a z-stack in the range of 15 μm varying the distance by 1 μm in the z-direction. The final image was acquired by adding all z-plane images into a single composite image at a certain region of interest.

For the SUP-B15 cell line, it is expected to see two distinct red and two green signals with the TEL/AML1 FISH probes. The presence of 2 red and 2 green signals (Figure S8A) were seen in >98% cells in agreement with the literature for this cell line. For the MLL break-apart probes, we would expect to see two red/green (or yellow) fusion signals and that was seen in most of the cells as noted from Figure S8B. Figure S9 shows the images of SUP-B15 cells FISH processed to identify the Philadelphia chromosome. Philadelphia chromosome is produced by the fusion of two genes in chromosome 9 and 22. Specifically, the translocation of ABL1 gene (9q34.11-q34.12, red) chromosome 9 and translocation BCR gene (22q11.22-q11.23, green) of chromosome 22 will result in Philadelphia translocation (t(9;22)(q34.12;q11.23),

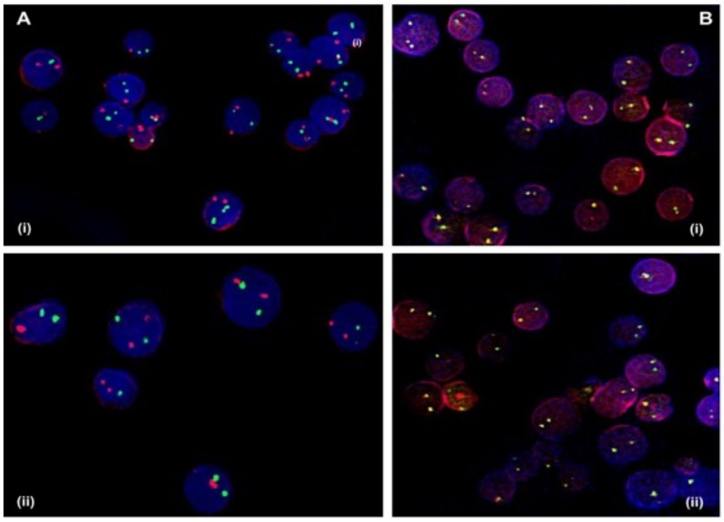

Figure S8. FISH processed images of SUP-B15 cell line showing signal for: (**A**) (i-ii) TEL/AML1 probe corresponding to TEL (ETV6, 12p13.2) region in red channel and AML1 (RUNX1, 21q22.12) region in the green channel. (**B**) (i-ii) MLL break-apart probe corresponding to MLL (KMT2A, 11q23.3) are shown (red and green channels). Images were acquired using a Keyence microscope and a Nikon 63x oil objective with DAPI, FITC (green) and Cy5 (red) filters.

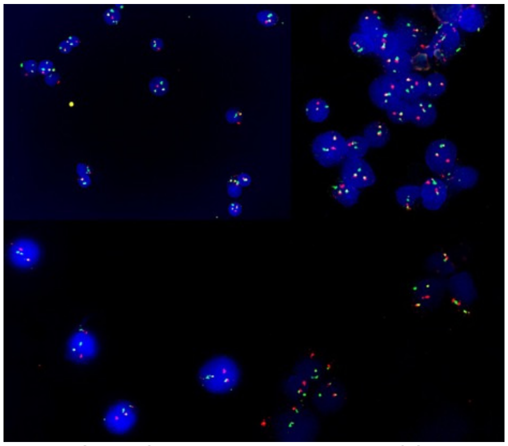

Figure S9. FISH processed images of SUP-B15 cell line showing signal for Philadelphia chromosome. Philadelphia chromosome is produced by the fusion of two genes in chromosome 9 and 22. Specifically, the translocation of ABL1 gene region (9q34.11-q34.12, red) chromosome 9 and translocation BCR gene region (22q11.22-q11.23, green) of chromosome 22 will result in Philadelphia translocation (t(9;22)(q34.12;q11.23), yellow). Images were acquired from Keyence microscope using Nikon 63x oil objective with DAPI, FITC and Cy5 filters.

yellow). As is evident from the image in Figure S9, there are yellow signals in >90% cells, which indicated the presence of the Philadelphia chromosome.

To process the images, z-stacks were loaded in all 3-channels and brightness and contrast were adjusted in the blue (DAPI), green, and red channels. Then, the full focus function was used to focus the 3-D projection of the z-stack to one single image. The processed images were then filtered through “haze reduction” and “black balance” filters to isolate the cell nuclei in the blue channel and to enhance the FISH signals in the green and red channels. DAPI processed images were then loaded to FIJI and the threshold was adjusted such that all the nuclei were clearly visible. The adjusted image was then used with Dilate (2X) and Watershed functions to clear out the single nuclei. After cell nuclei were separated, a mask was created and the green and red channel images were processed with the image calculator and the “AND” function to eliminate noise outside the cell nuclei. Afterwards, three channels were color shaded with the appropriate filter colors and images were overlaid to achieve the final processed images with FISH signal.

References

1. Nair, Soumya V., Malgorzata A. Witek, Joshua M. Jackson, Maria A. M. Lindell, Sally A. Hunsucker, Travis Sapp, Caroline E. Perry, Mateusz L. Hupert, Victoria Bae-Jump, Paola A. Gehrig, Weiya Z. Wysham, Paul M. Armistead, Peter Voorhees, and Steven A. Soper. "Enzymatic Cleavage of Uracil-Containing Single-Stranded DNA Linkers for the Efficient Release of Affinity-Selected Circulating Tumor Cells." *Chemical Communications* 51, no. 15 (2015): 3266-69.
2. Hupert, Mateusz L, Joshua M Jackson, Hong Wang, Małgorzata A Witek, Joyce Kamande, Matthew I Milowsky, Young E Whang, and Steven A Soper. "Arrays of High-Aspect Ratio Microchannels for High-Throughput Isolation of Circulating Tumor Cells (Ctcs)." *Microsystem technologies* 20, no. 10-11 (2014): 1815-25.
3. Gooding, R. P., A. Bybee, F. Cooke, A. Little, S. G. E. Marsh, E. Coelho, D. Gupta, D. Samson, and J. F. Apperley. "Phenotypic and Molecular Analysis of Six Human Cell Lines Derived from Patients with Plasma Cell Dyscrasia." *British journal of haematology* 106, no. 3 (1999): 669-81.
4. Kamande, Joyce, Maria Lindell, Maggie Ann Witek, Peter Voorhees, and Steven A. Soper. "Isolation of Circulating Plasma Cells from Blood of Patients Diagnosed with Clonal Plasma Cell Disorders Using Cell Selection Microfluidics." *Integrative Biology* 10, no. 2 (2018): 82-91.
